# Unprecedentedly targeted customization of molecular energy levels with auxiliary-groups in organic solar cell sensitizers†
†Electronic supplementary information (ESI) available: Synthesis and characterization, and additional photovoltaic data. See DOI: 10.1039/c5sc02778k


**DOI:** 10.1039/c5sc02778k

**Published:** 2015-10-09

**Authors:** Yongshu Xie, Wenjun Wu, Haibo Zhu, Jingchuan Liu, Weiwei Zhang, He Tian, Wei-Hong Zhu

**Affiliations:** a Key Laboratory for Advanced Materials and Institute of Fine Chemicals , Shanghai Key Laboratory of Functional Materials Chemistry , Collaborative Innovation Center for Coal Based Energy (i-CCE) , East China University of Science & Technology , Shanghai 200237 , China . Email: whzhu@ecust.edu.cn

## Abstract

In dye-sensitized solar cells (DSSCs), the HOMO–LUMO energy gap of organic sensitizers should be large enough to enable efficient electron injection and dye regeneration. However, the LUMOs of most practical organic dyes are always too high, making energy “waste”. In order to deepen the LUMOs, we focus on the targeted modulation of the molecular energy levels by embedding an electron donor or acceptor into the skeleton of a typical D–π–A model. The electron-rich group of 3,4-ethylenedioxythiophene (EDOT) lifts up the HOMO level with little influence on the LUMO, while the electron-deficient group of benzothiadiazole (BTD) or benzooxadiazole (BOD) mainly lowers the customized LUMO level. As a consequence, the auxiliary group change from EDOT (dye **WS-53**) to BOD (dye **WS-55**) brings forth a huge photoelectric conversion efficiency (PCE) increase by 38 fold from 0.24 to 9.46% based on an I^–^/I_3_^–^ redox couple, and even reaching a high PCE of 10.14% with **WS-55** under 0.3 sunlight irradiation.

## Abstract 2

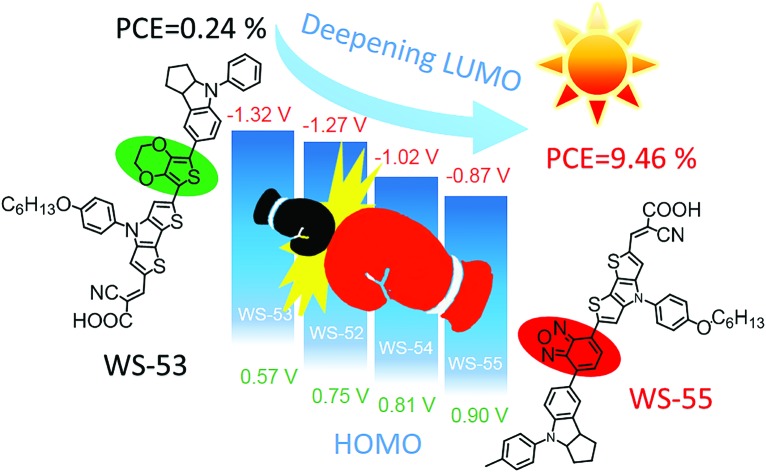
Lowering the LUMOs and decreasing energy “waste” is targeted through inserting an auxiliary group from an electron donor or acceptor into D–π–A organic sensitizers, and the photovoltaic efficiency increases 38 fold from 0.24 to 9.46%.

## Introduction

Dye sensitized solar cells (DSSCs) have received considerable attention due to their relatively high power conversion efficiency, low cost and high stability.^1^–^3^ Enormous research passion has also been devoted to metal-free organic dyes because of their excellent photophysical properties.^4^ Up to now, the donor–π bridge–acceptor (D–π–A) motif has been widely exploited for tailoring organic sensitizers.^5^ Among them, introducing auxiliary groups to the skeleton of the D–π–A system can exhibit a significant influence on the energy levels, light response, and dye stability as well as the photovoltaic performance of organic sensitizers.^6^,^7^

Generally, the LUMO and HOMO energy levels of organic sensitizers play important roles in electron injection and dye regeneration for DSSCs. Specifically, the driving force for electron injection from the excited dyes to the TiO_2_ conduction band (–0.5 V *vs.* NHE) should be greater than 0.2 V, and that for efficient dye regeneration from an iodine electrolyte (0.4 V *vs.* NHE) should be greater than 0.3 V.^8^ That is, the ideal LUMO and HOMO for an organic sensitizer should lie around –0.7 V and 0.7 V, respectively, with an appropriate band gap of about 1.4 eV. However, the LUMOs of most organic sensitizers actually used in DSSCs are too high, resulting in energy “waste”. Given the basic injection dynamics, lowering the LUMO level and lifting the HOMO level in organic dyes can be expected to narrow the band gap (*E*_0–0_) and extend the light response, thus efficiently optimizing the photovoltaic performance of DSSCs. In this regard, the targeted customization of molecular energy levels is still a challenge.

**Scheme 1:**
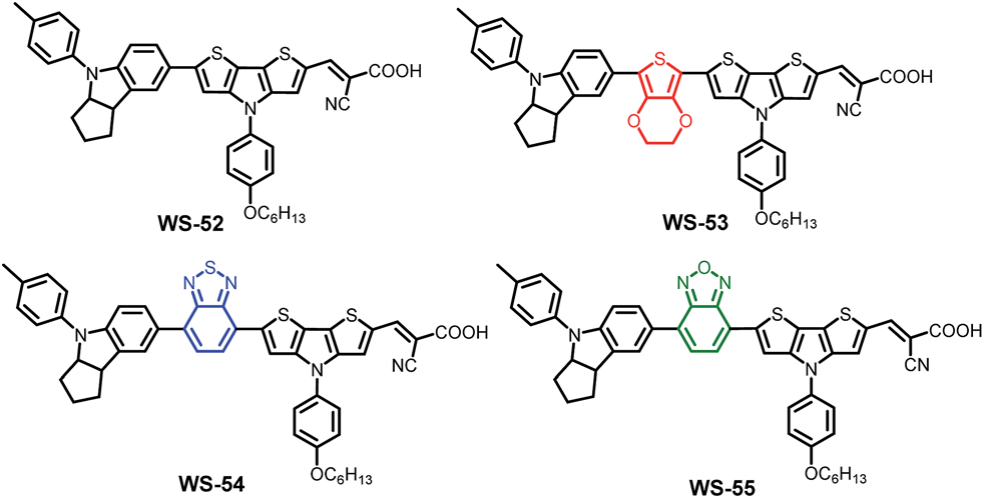
Molecular structures of dyes **WS-52**, **WS-53**, **WS-54** and **WS-55** containing different auxiliary groups for the targeted customization of molecular energy levels.

With this in mind, we herein present a new series of dithieno[3,2-*b*:2′,3′-*d*]pyrrole (DTP)-based organic sensitizers (Scheme 1) using different auxiliary groups in the π-bridge. Based on the reference dye **WS-52**, an electron-rich unit of 3,4-ethylenedioxythiophene (EDOT) and the electron-deficient groups of benzothiadiazole (BTD) and benzooxadiazole (BOD) were specifically introduced into **WS-53**, **WS-54** and **WS-55**, respectively. Interestingly, the embedding electron donor or acceptor can customize the molecular energy levels well. In distinct contrast with EDOT, the electron-deficient groups of BTD or BOD mainly lower the LUMO level, being capable of preventing the energy waste in electron injection. Typically, the auxiliary group change from EDOT (dye **WS-53**) to BOD (dye **WS-55**) induces a large PCE increase of 38 fold from 0.24% to 9.46% based on an I^–^/I_3_^–^ redox couple, even reaching a high PCE of 10.14% with **WS-55** under 0.3 sunlight irradiation.

## Results and discussion

**Fig. 1:**
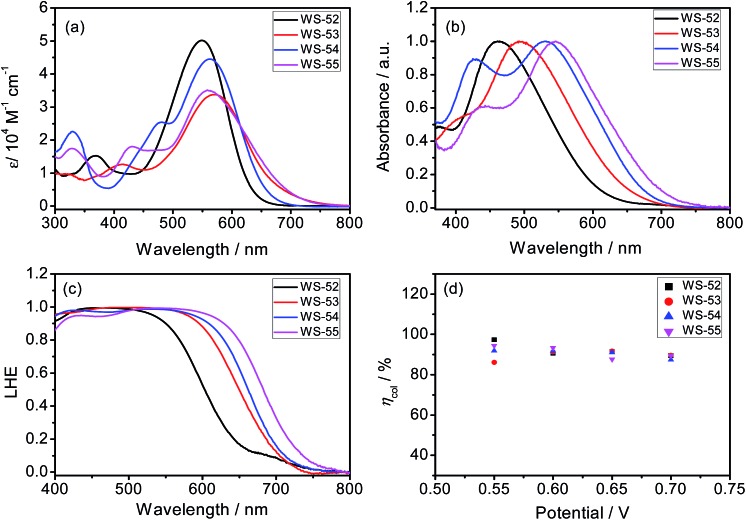
Absorption spectra of **WS-52**, **WS-53**, **WS-54** and **WS-55** in CH_2_Cl_2_ solution (a) and coated onto 3 μm TiO_2_ film (b), LHE spectra calculated from the absorption spectra of dye-loaded TiO_2_ film (c), and charge collection efficiency (*η*_coll_) in DSSCs as a function of bias potentials during the EIS measurement (d).

The syntheses of the dyes **WS-52**, **WS-53**, **WS-54** and **WS-55** are straightforward and described in the ESI.† Their absorption spectra in CH_2_Cl_2_ are depicted in Fig. 1a, and the corresponding data are summarized in Table 1. The reference dye **WS-52** shows two distinct absorption bands around 360 and 549 nm, corresponding to the π–π* and intramolecular charge transfer (ICT) transition bands, respectively. **WS-53** exhibits a significant bathochromic shift in the ICT band from 549 to 570 nm due to the extended π-conjugation with the EDOT unit. Through inserting the strong electron-withdrawing units of BTD and BOD, **WS-54** and **WS-55** exhibit bathochromic shifts of 14 and 9 nm, respectively. Compared with **WS-52**, the insertion of the auxiliary group (electron-rich or deficient group) into the π-spacer leads to an obvious red-shift of the ICT band in CH_2_Cl_2_. Upon adsorption onto TiO_2_ films (Fig. 1b), all four dyes show hypsochromic shifts due to the deprotonation of the cyanoacrylic acid group (Table 1). **WS-52** and **WS-53** show large hypsochromic shifts of 88 nm, from 549 to 461 nm, and 78 nm, from 570 to 492 nm, respectively. In contrast, **WS-54** and **WS-55**, containing strong electron-withdrawing auxiliary-groups, bestow much smaller hypsochromic shifts of 33 nm, from 563 to 530 nm, and 13 nm, from 558 to 545 nm, respectively. Obviously, upon the incorporation of strong electron withdrawing groups like BTD or BOD, the D–A–π–A featuring **WS-54** and **WS-55** bring forth a broader light response, which contributed to the smaller hypsochromic shift onto TiO_2_ and the presence of an additional sub-absorption band in the region of 400–450 nm.6*b*

**Table 1:** Photophysical and electrochemical properties of **WS-52**, **WS-53**, **WS-54** and **WS-55**, and their photovoltaic parameters for DSSCs

Dyes	*λ*_max_ ^*a*^ (nm)	*ε* ^*a*^ (M^–1^ cm^–1^)	*λ*_max_ ^*b*^ (nm)	HOMO^*c*^ (V)	*E*_0–0_ ^*d*^ (V)	LUMO^*e*^ (V)	*J*_SC_ (mA cm^–2^)	*V*_OC_ (mV)	FF	*η* ^*f*^ (%)
**WS-52**	549	50 160	461	0.75	2.02	–1.27	7.88 ± 0.06	640 ± 5	0.68 ± 0.01	3.44 ± 0.11
**WS-53**	570	33 788	492	0.57	1.89	–1.32	1.22 ± 0.12	440 ± 8	0.48 ± 0.03	0.24 ± 0.06
**WS-54**	563	44 514	530	0.81	1.83	–1.02	15.84 ± 0.06	660 ± 3	0.68 ± 0.01	7.14 ± 0.09
**WS-55**	558	35 105	545	0.90	1.77	–0.87	19.66 ± 0.47	678 ± 5	0.70 ± 0.02	9.46 ± 0.19
**WS-55** ^*g*^							6.74 ± 0.08	643 ± 3	0.73 ± 0.01	10.05 ± 0.09

^*a*^Absorption parameters were obtained in CH_2_Cl_2_.

^*b*^Absorption parameters were obtained on 3 μm nanocrystalline TiO_2_ film.

^*c*^The HOMO was obtained in CH_2_Cl_2_ with ferrocene (0.63 V *vs.* NHE) as an external reference.

^*d*^ *E*_0–0_ values were estimated from the wavelength at 10% maximum absorption intensity for the dye-loaded 3 μm nanocrystalline TiO_2_ film.

^*e*^The LUMO was calculated according to LUMO = HOMO – *E*_0–0_.

^*f*^The efficiency was obtained from the average value of five devices.

^*g*^The photovoltaic parameters were obtained under 0.3 sunlight irradiation.

**Fig. 2:**
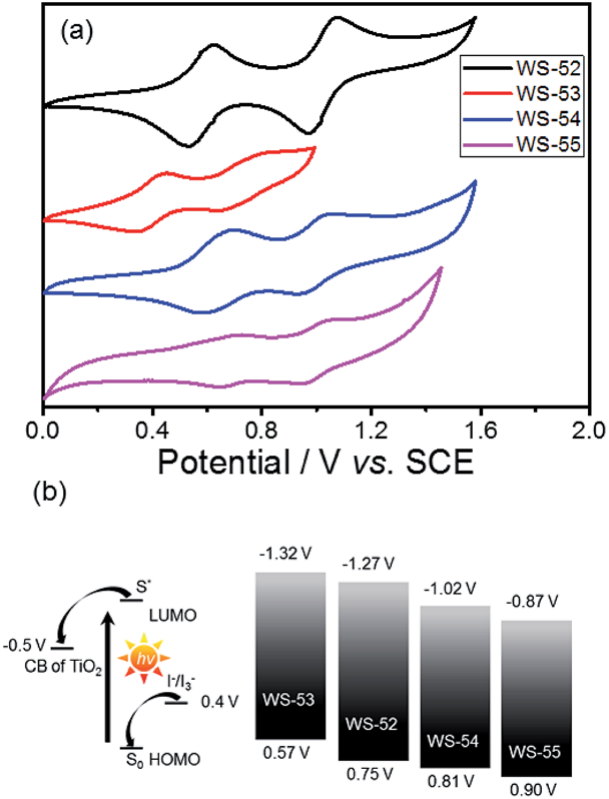
(a) Cyclic voltammograms of **WS-52**, **WS-53**, **WS-54** and **WS-55** in CH_2_Cl_2_, and (b) schematic diagram of the energy levels of the TiO_2_ conduction band, dyes, and I^–^/I_3_^–^ redox couple.

Next, we focus on the customized modulation of the molecular energy levels by embedding the auxiliary electron donor or acceptor into the skeleton of a typical D–π–A model. Based on the cyclic voltammetry measurements (Fig. 2a and Table 1), the first redox potentials corresponding to the HOMO values are 0.75, 0.57, 0.81 and 0.90 V (*vs.* NHE) for dyes **WS-52**, **WS-53**, **WS-54** and **WS-55**, respectively. Due to the electron donating property of EDOT, the HOMO of **WS-53** is lifted by 0.18 V with respect to **WS-52**, and there exists only a 0.17 V driving force for dye regeneration from the iodine electrolyte (Fig. 2b).8*b* As estimated from the HOMO and *E*_0–0_ (Table 1), the LUMO values of **WS-52**, **WS-53**, **WS-54** and **WS-55** are –1.27, –1.32, –1.02 and –0.87 V, respectively. Interestingly, the auxiliary electron-rich EDOT group predominantly lifts up the HOMO level with little influence on the LUMO, while the electron-deficient BTD or BOD group mainly lowers the LUMO level. It is noteworthy that the stronger electron-withdrawing capability of BOD in **WS-55** dramatically lowers the LUMO orbital from –1.27 V (**WS-52**) to –0.87 V. With regard to these four dyes, the insertion of different pull or push auxiliary groups can provide an efficient channel to realize the targeted customization of the HOMO and LUMO energy levels. Generally, the driving force for TiO_2_ electron injection is always much larger than the minimum requirement because the LUMOs of most organic sensitizers are always higher than –1.0 V. Comparing these four dyes, the customized LUMO orbital change from –1.27 V (reference **WS-52**) to –1.02 V (**WS-54**) to –0.87 V (**WS-55**) gives an unprecedented preferable modulation, in which we can efficiently decrease the “waste” in the electron-injection driving force, and thus efficiently decrease the HOMO–LUMO energy gap, resulting in a desirable light response in the long-wavelength range. Indeed, **WS-55** exhibited a long absorption onset wavelength as well as a promising PCE of 9.46% (Table 1), which is around 38 fold higher than the dye **WS-53** (0.24%). In the following, we obtain insight into how the incorporated auxiliary groups of EDOT, BTD and BOD play such a different role in the photovoltaic performance, especially focusing on the short-circuit current density (*J*_SC_) and open-circuit voltage (*V*_OC_).

**Fig. 3:**
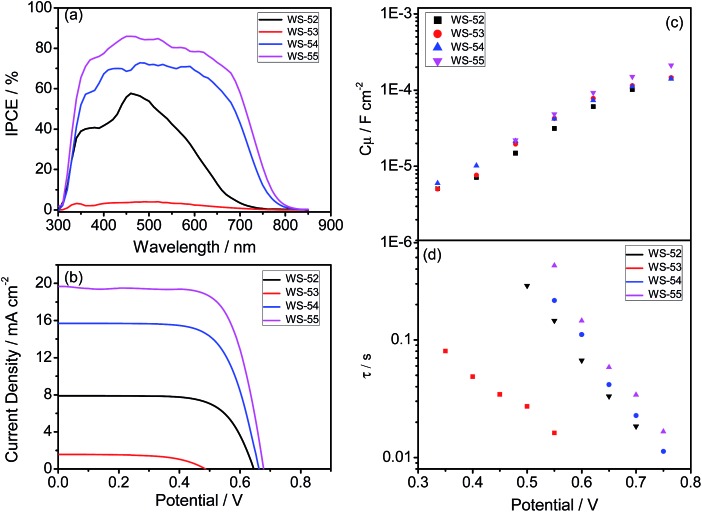
IPCE (a) and *J*–*V* curves (b), and bias potential against TiO_2_ capacitance (c) and electron lifetime (d) in DSSCs sensitized by **WS-52**, **WS-53**, **WS-54** and **WS-55**.

Generally, the photocurrent *J*_SC_ can be estimated from the incident photon-to-electron conversion efficiency (IPCE). Fig. 3a shows the IPCE curves as a function of the excitation wavelengths for these four dyes, which is critically dependent upon the inserted auxiliary group. To our great surprise, although the inserted EDOT unit can distinctly shift absorption to a long wavelength, **WS-53** exhibited very disappointing IPCE values (as low as 5%) across the whole visible range from 300–800 nm. In contrast, it is impressive that **WS-54** and **WS-55** bestow very broad and relatively high IPCE values. Upon increasing the electron-withdrawing capability of the auxiliary group, the IPCE onset wavelength was extended step by step (Fig. 3a), from 730 nm for the reference dye **WS-52** and 800 nm for **WS-54** to 840 nm for **WS-55**, which is very uncommon for pure organic sensitizers. Among these four dyes, **WS-55** also showed the highest IPCE plateau with a maximum value of 85.9%.

As is known, the IPCE value is determined on the basis of four factors as follows:5*a*

1 IPCE = LHE × *φ*_inj_ × *φ*_reg_ × *η*_coll_

where LHE is the light-harvesting efficiency related to the incident light absorbed by the dye molecules, *φ*_inj_ is the electron injection efficiency from the excited dye molecules into the TiO_2_ conduction band, *φ*_reg_ is the dye regeneration efficiency, and *η*_coll_ is the collection efficiency of the injected electrons to the FTO substrate. We looked into these four factors to explore the different IPCE behaviors. Initially, the LHE spectra were calculated from the absorption spectra of the dye-loaded TiO_2_ films (LHE = 1 – 10^–*α*^, where *α* is the intensity of the light absorption).5*a* As illustrated in Fig. 1c, the LHE curve for **WS-53** nearly reaches unity in the range of 400–600 nm, which is very similar to **WS-54** and **WS-55**. Obviously, the LHE effect on the IPCE characteristics is almost the same for **WS-53**, **WS-54** and **WS-55**. Based on previous extensive studies through femtosecond transient absorption spectroscopy, when the driving force for electron injection from the excited dyes to the nanoporous TiO_2_ conduction band (–0.5 V *vs.* NHE, Fig. S1†) is greater than 0.2 V, the injection rate for many organic dyes is much faster than the rate of luminescence decay, and therefore the *φ*_inj_ is always considered to be almost unity, and not the main handicap in the DSSC process.^9^ Obviously, here the energy differences between the LUMO and the TiO_2_ conduction band for all these dyes are also sufficient (>0.2 V), which can also guarantee the *φ*_inj_. Also from EIS analysis, their electron collection efficiencies are found to be similar, around 90% at a bias potential of 0.7 V (Fig. 1d).^10^ Thus, the remaining effect is the dye regeneration efficiency (*φ*_reg_). As shown in Fig. 2b, the HOMO energy levels for **WS-52**, **WS-54** and **WS-55** are 0.35, 0.41 and 0.50 V, which are more positive than the Nernst potential of the I^–^/I_3_^–^ electrolyte, respectively. All the driving forces are greater than 0.3 V, thus ensuring efficient dye regeneration. However, for **WS-53**, there existed only 0.17 V as a driving force for dye regeneration, which might heavily constrain the photocurrent to as low as 1.22 mA cm^–2^.

Moreover, based on the abovementioned eqn (1), assuming that the *φ*_inj_ is unity, the obtained *φ*_reg_*vs.* wavelength curves are shown in Fig. S2.† In the 480–640 nm visible region, the electron-deficient auxiliary groups (BTD or BOD) have a powerful effect on the *φ*_reg_, which almost reaches unity over 640 nm. With the enhancement of the electron-withdrawing capability, it is very advantageous to the regeneration of the oxidation state dyes, which makes the dye **WS-55** exhibit very good regeneration efficiency in this region, within 0.9–1. However, the electron-rich group EDOT makes the *φ*_reg_ of **WS-53** sharply drop with the increase in wavelength. This result is extremely consistent with the low driving force to dye regeneration (0.17 V) for **WS-53**, along with the very poor photocurrent (1.22 mA cm^–2^).

Apparently, among these four dyes, the insertion of the electron-donating EDOT unit undesirably lifts up the HOMO energy level, resulting in a detrimentally insufficient driving force for dye regeneration. In contrast, the incorporation of electron-withdrawing BTD and BOD units can dramatically lower or deepen the LUMO orbital levels, resulting in narrow HOMO–LUMO gaps with a preferable broad light response range. Given that BOD has stronger electron-withdrawing capability than BTD, we can decrease the LUMO orbital level step-by-step, and extend the light response range (Fig. 1b and 2b). In this way, upon the targeted modulation of the LUMO levels, the photocurrent *J*_SC_ for **WS-53**, **WS-52**, **WS-54** and **WS-55** increased stepwise from 1.22 to 7.88 to 15.84 to 19.66 mA cm^–2^ (Table 1), respectively, which corresponds well to the integrals of the IPCE curves (1.17, 7.10, 14.43 and 19.56 mA cm^–2^, Fig. S3†). In addition to the efficient level regulation action and high efficiency, the DSSCs based on **WS-55** also presented satisfactory photostability, remaining at 92% of the initial conversion efficiency after 500 h under visible-light irradiation (Fig. S4†).

Besides *J*_SC_, **WS-53** also exhibits a low photovoltage (*V*_OC_) of 440 mV, which is almost 200 mV lower than **WS-52**, **WS-54** and **WS-55**. As is known, the alternation of the photovoltage *V*_OC_ originates from a displacement of the electron quasi-Fermi-level (*E*_f_) in TiO_2_, which intrinsically stems from a change in the TiO_2_ conduction band edge (*E*_CB_) and/or a fluctuation in electron density (the charge recombination rate in DSSCs).^11^ Considering that the chemical capacitance (*C*_μ_) stands for the density of states in the bandgap of TiO_2_, we plot the variation of capacitance at different bias potentials with the fitting of electrochemical impedance spectra (EIS, Fig. S5†) for illustrating the shift in the *E*_CB_ of TiO_2_. Since these four dyes exhibited almost identical *C*_μ_ values (Fig. 3c), we can rule out a shift in the TiO_2_ conduction band as the main reason for the rather low photovoltage of **WS-53**. On the other hand, the fluctuation in TiO_2_ electron density can also induce a difference in *V*_OC_, which is closely related to the recombination resistance.^12^Fig. 3d illustrates the plots of the electron lifetime under a series of potential biases, and the calculated electron lifetimes lie in the order of **WS-55** > **WS-54** > **WS-52** > **WS-53**, which is exactly consistent with the sequence of *V*_OC_. Among these four dyes, the targeted LUMO and HOMO energy levels of **WS-55** are the most desirable. As a matter of fact, the change of auxiliary groups can distinctly increase the photovoltaic efficiency from EDOT (dye **WS-53**, 0.24 ± 0.06%) to BDT (dye **WS-54**, 7.14 ± 0.09%) to BOD (dye **WS-55**, 9.46 ± 0.19%, obtained from the average of five cells listed in Fig. S6†). Under 0.3 sunlight irradiation, **WS-55** can even achieve a high PCE of 10.14% (Table 1 and Fig. S7†).

## Conclusions

In summary, we have unprecedentedly targeted the customization of molecular energy levels by introducing auxiliary groups from an electron donor or acceptor into D–π–A featuring organic sensitizers. Dyes **WS-52**, **WS-53**, **WS-54** and **WS-55** exhibit well-modulated molecular energy levels in a stepwise manner, thus distinctly lowering the LUMOs, which are always too high and make energy “waste” in most practical organic dyes. As demonstrated, the photovoltaic efficiency is greatly improved when changing the auxiliary group from EDOT (dye **WS-53**, 0.24%) to BDT (dye **WS-54**, 7.14%) or BOD (dye **WS-55**, 9.46%). Moreover, **WS-55** can even achieve a high PCE of 10.14% under 0.3 sunlight irradiation. It shows how the delicate balance of LUMO energy levels and HOMO–LUMO energy gaps guarantees the optimizing of photovoltaic properties.

## Supplementary Material

Supplementary information
