# Supplementary material for: Unprecedentedly targeted customization of molecular energy levels with auxiliary-groups in organic solar cell sensitizers
Source: Chem Sci. 2015 Oct 9;7(1):544–9. doi: 10.1039/c5sc02778k (PMC5952894; doi:10.1039/c5sc02778k)
Supplement: Supplementary file 1 [file SC-007-C5SC02778K-s001.pdf]

Electronic Supplementary Material (ESI) for Chemical Science.
This journal is © The Royal Society of Chemistry 2015

Electronic Supplementary Information (ESI)

Unprecedentedly targeted customization of molecular energy levels with auxiliary-group in organic solar cell sensitizers†

Yongshu Xie,‡ Wenjun Wu,‡ Haibo Zhu, Jingchuan Liu, Weiwei Zhang, He Tian and Wei-Hong Zhu*

[a] *Key Laboratory for Advanced Materials and Institute of Fine Chemicals, Shanghai Key Laboratory of Functional Materials Chemistry, Collaborative Innovation Center for Coal Based Energy (i-CCE), East China University of Science and Technology, Shanghai 200237, P. R. China. Fax:(+86)21-6425-2758.E-mail: whzhu@ecust.edu.cn.*

‡ These authors contributed equally to this work.

Contents

1. **Materials and reagents**
2. **Synthesis and characterization**
3. **Fabrication of dye-sensitized solar cells**
4. **Photovoltaic characterization**
5. **Electrochemical impedance spectroscopy (EIS)**
6. **SEM spectra of TiO2 film**
7. **IPCE spectra of WS-52, WS-53, WS-54 and WS-55, and their corresponding integrated current curves**
8. **The device stability character of dye WS-55**
9. **The electrochemical impedance spectroscopy**
10. **Photovoltaic data of different DSSC samples based on WS-55**
11. **Photoelectric parameters (J_{SC}, V_{OC} and PCE) under different light intensity based on Dye**
12. **^{1}H NMR, ^{13}C NMR and HRMS of intermediates and targeted dyes**

1. Materials and reagents

^{1}H and ^{13}C NMR spectra were recorded on a Bruker AM 400 spectrometer with tetramethylsilane (TMS) as an internal standard. High resolution mass spectrometry (HRMS) was performed using a Waters LCT Premier XE spectrometer. The UV-Vis spectra were recorded with a Varian Cary 100 spectrophotometer. Cyclic voltammograms were performed with a Versastat II electrochemical workstation (Princeton Applied Research) using a normal three-electrode cell with a Pt working electrode, Pt wire counter electrode, and regular calomel reference electrode in a saturated KCl solution. An amount of 0.1 M tetrabutylammonium hexafluorophosphate (TBAPF6) was used as the supporting electrolyte in CH_2Cl_2.
The starting materials **1**,[1] **2**[1] and **6**[2] were prepared according to our previous reports. The intermediate **3**[3] and **7**[3] were synthesized via reported methods. THF was pre-dried over 4 Å molecular sieves and distilled under argon atmosphere from sodium benzophenoneketyl immediately prior to use. All other solvents and chemicals used in this work were of reagent grade and used without further purification.

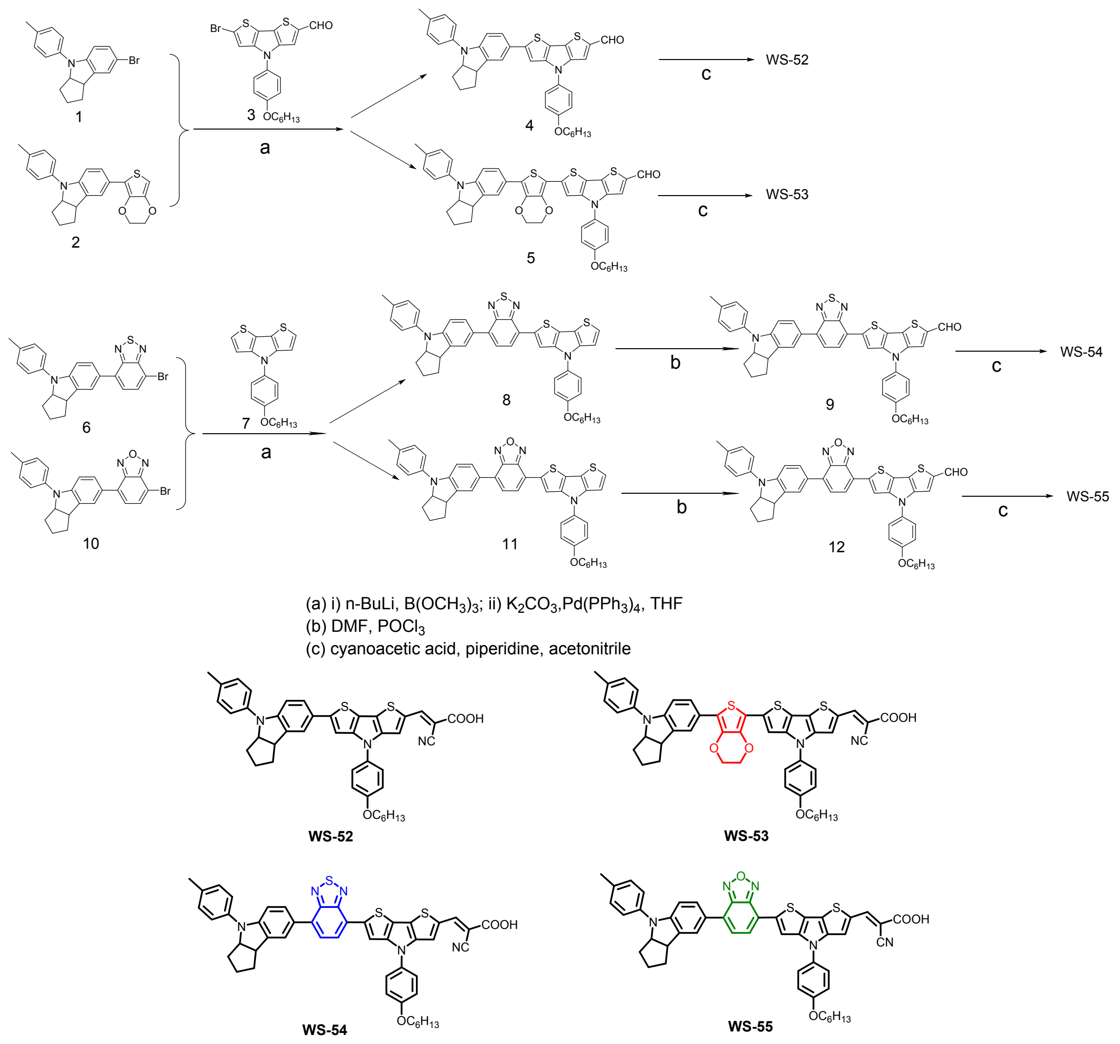

Scheme 1. Synthetic route of target dyes **WS-52**, **WS-53**, **WS-54** and **WS-55** containing different auxiliary groups for targeted customization of molecular energy levels.

2. Synthesis and characterization

2.1 Synthesis of compound 4.

n-BuLi (0.8 mL, 1.92 mmol) was added drop-wise into compound **1** (520 mg, 1.58 mmol) dissolved in dry THF (15 mL) at -78 °C under argon. The solution was stirred at the same temperature for 1 h, and $B(OCH_3)_3$ (240 mg, 2.31 mmol) was added. After stirring at the temperature for 4 h, the mixture was gradually warmed to room temperature and used for the next Suzuki reaction without purification. To a three-neck round-bottom flask, the previous mixture, compound **3** (263 mg, 0.57 mmol), 30 mL THF, 15 mL 2 M K_2CO_3 aqueous solution and $Pd(PPh_3)_4$ (40 mg, 0.03 mmol) were added. The mixture was refluxed for 8 h. After cooling to room temperature, the crude compound was extracted into ethyl acetate (30 mL × 3), washed with brine and water, and dried over anhydrous sodium sulfate. After removing solvent under reduced pressure, the residue was purified by column chromatography (petroleum/CH_2Cl_2 = 1/1) on silica gel to yield a red powder 186 mg, yield 51.7%. ^1H NMR (400 MHz, $CDCl_3$, ppm): δ 9.79 (s, 1 H), 7.63 (s, 1 H), 7.46 (d, J = 8.8 Hz, 2 H), 7.35 (s, 1 H), 7.30 (dd, J_1 = 8.3 Hz, J_2 = 1.8 Hz, 1 H), 7.12–7.20 (m, 4 H), 7.10 (s, 1 H), 7.06 (d, J = 8.7 Hz, 2 H), 6.84 (d, J = 8.3 Hz, 1 H), 4.76-4.85 (m, 1 H), 4.03 (t, J = 6.5 Hz, 2 H), 3.78-3.87 (m, 1 H), 2.34 (s, 3 H), 1.99-2.12 (m, 1 H), 1.86–1.96 (m, 2 H), 1.73-1.86 (m, 3 H),1.63-1.72 (m, 1 H), 1.45-1.59 (m, 3 H), 1.32-1.41 (m, 4 H), 0.90-0.96 (m, 3 H). ^{13}C NMR (100 MHz, $CDCl_3$, ppm): δ 182.68, 158.16, 149.55, 149.46, 148.53, 143.22, 139.87, 139.47, 135.76, 131.96, 131.57, 129.86, 125.46, 125.19, 124.81, 124.78, 122.19, 120.40, 115.68, 113.73, 107.42, 104.94, 69.32, 68.51, 45.25, 35.15, 33.65, 31.61, 29.23, 25.76, 24.43, 22.65, 20.83, 14.08.

2.2 Synthesis of compound WS-52.

To a two-neck round-bottom flask, compound **4** (136 mg, 0.22 mmol), piperidine (0.5 mL), cyanoacetic acid (148 mg, 1.76 mmol), 20 mL acetonitrile were added. The mixture was heated at 80 °C for 8 h under argon. The crude product was extracted with CH_2Cl_2 (30 mL × 3), washed with water, and dried

over anhydrous $MgSO_4$. The solvent was removed by rotary evaporation under reduced pressure. The crude product was purified by column chromatography with CH_3OH/CH_2Cl_2 (1/10, v/v) as eluent on silica gel to afford **WS-52** as a deep red solid 96 mg, yield 62.7%. ^1H NMR (400 MHz, DMSO, ppm): δ 13.35 (s, 1 H), 8.48 (s, 1 H), 8.07 (s, 1 H), 7.61 (d, J = 8.8 Hz, 2 H), 7.57 (s, 1 H), 7.46 (s, 1 H), 7.38 (dd, J_1 = 8.4 Hz, J_2 = 1.6 Hz, 1 H), 7.17–7.23 (m, 4 H), 7.15 (d, J = 8.9 Hz, 2 H), 6.83 (d, J = 8.4 Hz, 1 H), 4.84-4.92 (m, 1 H), 4.05 (t, J = 6.4 Hz, 2 H), 3.78-3.87 (m, 1 H), 2.28 (s, 3 H), 1.94-2.11 (m, 1 H), 1.67-1.88 (m, 5 H), 1.55-1.67 (m, 1 H), 1.40-1.51 (m, 2 H), 1.29-1.39 (m, 5 H), 0.86-0.90 (m, 3 H). ^{13}C NMR (100 MHz, DMSO, ppm): δ 164.38, 157.48, 149.65, 149.33, 147.76, 142.69, 139.22, 135.65, 132.46, 131.08, 129.76, 125.67, 125.22, 124.52, 124.26, 123.17, 121.97, 119.82, 117.45, 115.60, 113.24, 106.86, 105.40, 99.32, 68.33, 67.84, 44.40, 34.80, 33.00, 30.99, 28.61, 25.19, 23.92, 22.08, 20.37, 13.91. HRMS–ESI (*m/z*): [M^+] Calcd. for ($C_{42}H_{39}N_3O_3S_2$), 697.2433, found: 697.2439.

2.3 Synthesis of compound 5.

n-BuLi (0.6 mL, 1.44 mmol) was added drop-wise into compound 1 (460 mg, 1.18 mmol) dissolved in dry THF (15 mL) at -78 °C under argon. The solution was stirred at the same temperature for 1 h, and $B(OCH_3)_3$ (180 mg, 1.73 mmol) was added. After stirring at the temperature for 4 h, the mixture was gradually warmed to room temperature and used for the next Suzuki reaction without purification. To a three-neck round-bottom flask, the previous mixture, compound 3 (456 mg, 0.98 mmol), 30 mL THF, 15 mL 2 M K_2CO_3 aqueous solution and $Pd(PPh_3)_4$ (40 mg, 0.03 mmol) were added. The mixture was refluxed for 8 h. After cooling to room temperature, the crude compound was extracted into ethyl acetate (30 mL × 3), washed with brine and water, and dried over anhydrous sodium sulfate. After removing solvent under reduced pressure, the residue was purified by column chromatography (petroleum/CH_2Cl_2 = 1/1) on silica gel to yield a red powder 128 mg, yield 28.2%. ^1H NMR (400 MHz, $CDCl_3$, ppm): δ 9.78 (s, 1 H), 7.61 (s, 1 H), 7.46 (s, 1 H), 7.44 (s, 2

H), 7.39 (d, J = 8.0 Hz, 1 H), 7.13–7.18 (m, 4 H), 7.10 (s, 1 H), 7.04 (d, J = 8.8 Hz, 2 H), 6.86 (d, J = 8.4 Hz, 1 H), 4.69-4.83 (m, 1 H), 4.40 (d, J = 4.2 Hz, 2 H), 4.34 (d, J = 4.4 Hz, 2 H), 4.03 (t, J = 6.4 Hz, 2 H), 3.73-3.87 (m, 1 H), 2.33 (s, 3 H), 1.98-2.10 (m, 1 H), 1.73–1.95 (m, 5 H), 1.60-1.70 (m, 1 H), 1.46-1.55 (m, 3 H), 1.33-1.42 (m, 4 H), 0.82-0.90 (m, 3 H). ^{13}C NMR (100 MHz, $CDCl_3$, ppm): δ 182.61, 158.12, 148.76, 147.18, 143.56, 140.16, 139.64, 139.23, 139.03, 136.47, 135.37, 131.51, 131.46, 129.77, 125.76, 124.93, 124.72, 122.69, 122.52, 119.98, 117.80, 115.66, 114.50, 108.30, 107.48, 105.48, 69.11, 68.48, 65.05, 64.58, 45.36, 35.08, 33.72, 31.59, 29.22, 25.75, 24.41, 22.63, 20.79, 14.07. HRMS–ESI (*m/z*): [M^+] Calcd. for ($C_{45}H_{42}N_2O_4S_3$), 770.2307, found: 770.2307.

2.4 Synthesis of compound WS-53.

To a two-neck round-bottom flask, compound **5** (112 mg, 0.14 mmol), piperidine (0.5 mL), cyanoacetic acid (118 mg, 1.40 mmol), 20 mL acetonitrile were added. The mixture was heated at 80 °C for 8 h under argon. The crude product was extracted with CH_2Cl_2 (30 mL × 3), washed with water, and dried over anhydrous $MgSO_4$. The solvent was removed by rotary evaporation under reduced pressure. The crude product was purified by column chromatography with CH_3OH/CH_2Cl_2 (1/10, v/v) as eluent on silica gel to afford **WS-53** as a deep red solid 73 mg, yield 62.4%. ^{1}H NMR (400 MHz, DMSO, ppm): δ 8.45 (s, 1 H), 8.04 (s, 1 H), 7.59 (d, J = 8.7 Hz, 2 H), 7.45 (s, 1 H), 7.36 (d, J = 7.9 Hz, 1 H), 7.11-7.25 (m, 7 H), 6.85 (d, J = 8.6 Hz, 1 H), 4.81-4.90 (m, 1 H), 4.45 (s, 2 H), 4.40 (s, 2 H), 4.07 (t, J = 6.3 Hz, 2 H), 3.76-3.88 (m, 1 H), 2.28 (s, 3 H), 1.94-2.01 (m, 1 H), 1.83–1.68 (m, 5 H), 1.57-1.66 (m, 1 H), 1.40-1.51 (m, 3 H), 1.30-1.39 (m, 4 H), 0.88-0.94 (m, 3 H). HRMS–ESI (*m/z*): [M^+] Calcd. for ($C_{48}H_{43}N_3O_5S_3$), 837.2365, found: 837.2369.

2.5 Synthesis of compound 8.

To a solution of compound **7** (480 mg, 1.35 mmol) in dry THF (20 mL), n-BuLi (0.6 mL, 1.44 mmol) was added drop wise at -78 °C under argon. The solution was stirred at the same temperature for 1 h, and $B(OCH_3)_3$ (196 mg,

1.89 mmol) was added. After stirring at the temperature for 4 h, the mixture was gradually warmed to room temperature and used for the next Suzuki reaction without purification. The previous mixture was reacted with **6** (870 mg, 1.88 mmol) under Suzuki coupling reaction conditions using $Pd(PPh_3)_4$ (20 mg, 0.017 mmol) and a 2 M K_2CO_3 (20 mL) aqueous solution in THF (40 mL) solution at 80 °C for 10 h. After cooling to room temperature, the crude compound was extracted into ethyl acetate (30 mL × 3), washed with brine and water, and dried over anhydrous sodium sulfate. After removing solvent under reduced pressure, the residue was purified by column chromatography (petroleum/CH_2Cl_2 = 4/1) on silica gel to yield a red powder 473 mg, 47.6%. ^1H NMR (400 MHz, $CDCl_3$, ppm): δ 8.31 (s, 1 H), 7.88 (d, J = 7.6 Hz, 1 H), 7.76 (s, 1 H), 7.71 (dd, J_1 = 8.3 Hz, J_2 = 1.7 Hz, 1 H), 7.64 (d, J = 7.6 Hz, 1 H), 7.55 (d, J = 8.8 Hz, 2 H), 7.23 (s, 1 H), 7.20 (d, J = 5.4 Hz, 1 H), 7.17 (d, J = 8.4 Hz, 2 H), 7.08-7.11 (m, 2 H), 7.07 (s, 1 H), 7.03 (d, J = 8.3 Hz, 2 H), 4.82-4.90 (m, 1 H), 4.05 (t, J = 6.4 Hz, 2 H), 3.89-3.98 (m, 1 H), 2.35 (s, 3 H), 2.03-2.15 (m, 1 H), 1.91–2.01 (m, 2 H), 1.75-1.90 (m, 3 H), 1.64-1.74 (m, 1 H), 1.57-1.63 (m, 1 H), 1.46-1.55 (m, 2 H), 1.32-1.44 (m, 4 H), 0.90-0.98 (m, 3 H). ^{13}C NMR (100 MHz, $CDCl_3$, ppm): δ 157.65, 154.25, 152.76, 148.34, 145.11, 140.18, 137.66, 135.36, 132.75, 132.60, 131.62, 129.81, 128.76, 127.28, 126.27, 125.83, 125.49, 125.13, 124.54, 124.27, 120.24, 116.61, 116.48, 115.54, 112.60, 112.02, 107.45, 69.27, 68.44, 45.45, 35.21, 33.75, 31.64, 29.30, 25.80, 24.48, 22.67, 20.84, 14.11. HRMS–ESI (*m/z*): $[M+H]^+$ Calcd. for ($C_{44}H_{41}N_4OS_3$), 737.2443, found: 737.2441.

2.6 Synthesis of compound 9.

$POCl_3$ (210 mg, 1.37 mmol) was added in dry DMF (10 mL) at 0 °C. The dry DMF (20 mL) solution of compound **8** (448 mg, 0.61 mmol) was added via a syringe. After stirred for 30 min, the mixture was stirred at room temperature overnight. Ice water was added to terminate the reaction. After neutralization with Na_2CO_3 solution to pH 8-10, the mixture was extracted by CH_2Cl_2 (30 mL

× 3). The combined organic layer was washed with H_2O and brine, dried over Na_2SO_4, and evaporated under reduced pressure. The residue was purified by column chromatography (petroleum/CH_2Cl_2 = 1/1) on silica gel to yield a red powder 365 mg, yield 78.3%. ^{1}H NMR (400 MHz, $CDCl_3$, ppm): δ 9.84 (s, 1 H), 8.26 (s, 1 H), 7.90 (d, J = 7.6 Hz, 1 H), 7.77 (s, 1 H), 7.71 (dd, J_1 = 8.4 Hz, J_2 = 1.5 Hz, 1 H), 7.68 (s, 1 H), 7.64 (d, J = 7.6 Hz, 1 H), 7.53 (d, J = 8.8 Hz, 2 H), 7.24 (d, J = 8.4 Hz, 2 H), 7.18 (d, J = 8.3 Hz, 2 H), 7.11 (d, J = 8.8 Hz, 2 H), 7.01 (d, J = 8.4 Hz, 1 H), 4.82-4.91 (m, 1 H), 4.06 (t, J = 6.5 Hz, 2 H), 3.88-3.97 (m, 1 H), 2.35 (s, 3 H), 2.03-2.16 (m, 1 H), 1.91–2.01 (m, 2 H), 1.75-1.90 (m, 3 H), 1.64-1.74 (m, 1 H), 1.58-1.63 (m, 1 H), 1.46-1.55 (m, 2 H), 1.32-1.44 (m, 4 H), 0.91-0.98 (m, 3 H). ^{13}C NMR (100 MHz, $CDCl_3$, ppm): δ 182.93, 158.26, 154.12, 152.65, 149.09, 148.64, 144.24, 143.00, 140.88, 140.00, 135.43, 133.98, 131.82, 131.44, 129.84, 128.95, 126.89, 126.20, 125.92, 125.53, 124.81, 124.45, 120.38, 116.15, 115.76, 111.86, 107.38, 69.29, 68.52, 45.40, 35.25, 33.69, 31.63, 29.26, 25.79, 24.46, 22.67, 20.86, 14.11. HRMS–ESI (*m/z*): $[M+H]^+$ Calcd. for ($C_{45}H_{41}N_4O_2S_3$), 765.2392, found: 765.2388.

2.7 Synthesis of compound WS-54.

To a two-neck round-bottom flask, compound **9** (342 mg, 0.45 mmol), piperidine (0.5 mL), cyanoacetic acid (335 mg, 3.99 mmol), 20 mL acetonitrile were added. The mixture was heated at 80 °C for 8 h under argon. The crude product was extracted with CH_2Cl_2 (30 mL × 3), washed with water, and dried over anhydrous $MgSO_4$. The solvent was removed by rotary evaporation under reduced pressure. The crude product was purified by column chromatography with CH_3OH/CH_2Cl_2 (1/10, v/v) as eluent on silica gel to afford **WS-54** as a deep red solid 264 mg, yield 70.6%. ^{1}H NMR (400 MHz, DMSO, ppm): δ 8.37 (s, 1 H), 8.10 (s, 1 H), 7.95 (m, 2 H), 7.77 (s, 1 H), 7.65 (t, J = 7.6 Hz, 2 H), 7.53 (d, J = 8.5 Hz, 2 H), 7.18 (m, 4 H), 7.12 (d, J = 8.8 Hz, 2 H), 6.86 (d, J = 8.4 Hz, 1 H), 4.81-4.90 (m, 1 H), 4.04 (t, J = 6.3 Hz, 2 H), 3.78-3.87 (m, 1 H), 2.28 (s, 3 H), 1.88-2.11 (m, 2 H), 1.68-1.87 (m, 5 H), 1.53-1.67 (m, 1 H), 1.40-

1.50 (m, 2 H), 1.28-1.39 (m, 4 H), 0.90 (t, J = 6.9 Hz, 3 H). ^{13}C NMR (100 MHz, $CDCl_3$, ppm): δ 164.14, 157.49, 152.98, 151.70, 148.06, 147.51, 143.56, 142.68, 139.39, 134.93, 134.13, 132.40, 130.81, 130.68, 129.90, 129.73, 128.69, 126.30, 126.09, 125.69, 125.16, 124.41, 123.68, 121.28, 119.58, 116.46, 115.59, 110.83, 106.70, 68.28, 67.86, 44.52, 34.85, 33.07, 28.66, 25.21, 23.94, 22.10, 20.37, 13.93. HRMS–ESI (*m/z*): $[M+H]^+$ Calcd. for ($C_{48}H_{42}N_5O_3S_3$), 832.2450, found: 832.2438.

2.8 Synthesis of compound 11.

To a solution of compound **7** (440 mg, 1.24 mmol) in dry THF (30 mL), n-BuLi (0.6 mL, 1.44 mmol) was added drop wise at -78 °C under argon. The solution was stirred at the same temperature for 1 h, and $B(OCH_3)_3$ (212 mg, 2.04 mmol) was added. After stirring at the temperature for 4 h, the mixture was gradually warmed to room temperature and used for the next Suzuki reaction without purification. The previous mixture was reacted with **10** (375 mg, 0.84 mmol) under Suzuki coupling reaction conditions using $Pd(PPh_3)_4$ (20 mg, 0.017 mmol) and a 2 M K_2CO_3 (20 mL) aqueous solution in THF (30 mL) solution at 80 °C for 10 h. After cooling to room temperature, the crude compound was extracted into ethyl acetate (30 mL × 3), washed with brine and water, and dried over anhydrous sodium sulfate. After removing solvent under reduced pressure, the residue was purified by column chromatography (petroleum/CH_2Cl_2 = 4/1) on silica gel to yield a red powder 256 mg, 42.3%. ^1H NMR (400 MHz, $CDCl_3$, ppm): δ 8.21 (s, 1 H), 7.85 (s, 1 H) , 7.78 (dd, J_1 = 8.4 Hz, J_2 = 1.7 Hz, 1 H), 7.62 (d, J = 7.5 Hz, 1 H), 7.53 (d, J = 8.8 Hz, 2 H), 7.49 (d, J = 7.5 Hz, 1 H), 7.23 (d, J = 3.4 Hz, 1 H), 7.22 (s, 2 H), 7.18 (d, J = 8.4 Hz, 2 H), 7.08-7.10 (m, 3 H), 6.98 (d, J = 8.4 Hz, 1 H), 4.87 (m, 1 H), 4.06 (t, J = 6.5 Hz, 2 H), 3.92 (m, 1 H), 2.35 (s, 3 H), 2.06-2.12 (m, 1 H), 1.94-1.97 (m, 2 H), 1.79-1.89 (m, 3 H), 1.67-1.71 (m, 1 H), 1.58-1.63 (m, 1 H), 1.50-1.52 (m, 2 H), 1.37-1.40 (m, 4 H), 0.92-0.96 (m, 3 H). ^{13}C NMR (100 MHz, $CDCl_3$, ppm): δ 157.83, 149.21, 149.05, 148.19, 145.49, 145.18, 139.81, 135.91, 135.70,

132.28, 132.08, 129.86, 128.00, 127.81, 125.72, 125.50, 125.19, 124.84, 124.58, 124.52, 121.56, 120.58, 116.34, 115.65, 113.67, 112.05, 107.49, 69.38, 68.46, 45.34, 35.27, 33.64, 31.61, 29.27, 25.77, 24.42, 22.65, 20.84, 14.07.

2.9 Synthesis of compound 12.

$POCl_3$ (182 mg, 1.19 mmol) was added in dry DMF (10 mL) at 0 °C. The dry DMF (20 mL) solution of compound **11** (240 mg, 0.33 mmol) was added via a syringe. After stirred 30 min, the mixture was stirred at room temperature overnight. Ice water was added to terminate the reaction. After neutralization with Na_2CO_3 solution to pH 8-10, the mixture was extracted by CH_2Cl_2 (30 mL × 3). The combined organic layer was washed with H_2O and brine, dried over Na_2SO_4, and evaporated under reduced pressure. The residue was purified by column chromatography (petroleum/CH_2Cl_2 = 1/1) on silica gel to yield a red powder 156 mg, yield 63.2%. ^{1}H NMR (400 MHz, $CDCl_3$, ppm): δ 9.87 (s, 1 H), 8.18 (s, 1 H), 7.86 (s, 1 H) , 7.79 (dd, J_1 = 8.4 Hz, J_2 = 1.8 Hz, 1 H), 7.70 (s, 1 H), 7.67 (d, J = 7.5 Hz, 1 H), 7.52 (m, 3 H), 7.22 (d, J = 8.7 Hz, 2 H), 7.18 (d, J = 8.6 Hz, 2 H), 7.12 (d, J = 8.8 Hz, 2 H), 6.96 (d, J = 8.5 Hz, 1 H), 4.88 (m, 1 H), 4.07 (t, J = 6.5 Hz, 2 H), 3.92 (m, 1 H), 2.36 (s, 3 H), 2.06-2.12 (m, 1 H), 1.80-1.87 (m, 2 H), 1.75-1.80 (m, 2 H), 1.70-1.73 (m, 1 H), 1.67-1.70 (m, 1 H), 1.59-1.63 (m, 1 H), 1.49-1.53 (m, 2 H), 1.39-1.42 (m, 4 H), 0.93-0.96 (m, 3 H). ^{13}C NMR (100 MHz, $CDCl_3$, ppm): δ 182.93, 158.41, 149.37, 149.07, 148.99, 147.98, 144.55, 141.35, 140.99, 139.58, 135.78, 132.27, 131.14, 129.88, 129.16, 128.25, 126.95, 125.18, 124.81, 124.74, 124.55, 124.04, 120.69, 120.46, 115.86, 115.72, 112.96, 107.41, 69.38, 68.53, 45.27, 35.30, 33.56, 31.60, 29.23, 25.76, 24.39, 22.65, 20.86, 14.08. HRMS–ESI (*m/z*): $[M+H]^+$ Calcd. for ($C_{45}H_{41}N_4O_3S_2$), 749.2620, found: 749.2628.

2.10 Synthesis of compound WS-55.

To a two-neck round-bottom flask, compound **12** (132 mg, 0.18 mmol), piperidine (0.5 mL), cyanoacetic acid (257 mg, 3.13 mmol), 40 mL acetonitrile

were added. The mixture was heated at 80 °C for 8 h under argon. The crude product was extracted with CH_2Cl_2 (30 mL × 3), washed with water, and dried over anhydrous $MgSO_4$. The solvent was removed by rotary evaporation under reduced pressure. The crude product was purified by column chromatography with CH_3OH/CH_2Cl_2 (1/10, v/v) as eluent on silica gel to afford **WS-55** as a deep red solid 102 mg, yield 69.5%. ^1H NMR (400 MHz, DMSO+$CDCl_3$, ppm): δ 8.08 (s, 1 H), 8.00 (s, 1 H), 7.79 (m, 2 H), 7.70 (s, 1 H), 7.66 (m, 2 H), 7.59 (m, 2 H), 7.19 (m, 6 H), 6.88 (m, 1 H), 4.88 (m, 1 H), 4.09 (t, J = 6.2 Hz, 2 H), 3.84 (m, 1 H), 2.30 (s, 3 H), 1.97-2.03 (m, 2 H), 1.77-1.82 (m, 5 H), 1.58-1.67 (m, 1 H), 1.50 (m, 2 H), 1.35-1.37 (m, 4 H), 0.92 (t, J = 7.0 Hz, 3 H). HRMS–ESI (*m/z*): $[M-H]^-$ Calcd. for ($C_{48}H_{40}N_5O_4S_2$), 814.2522, found: 814.2525.

3. Fabrication of dye-sensitized solar cells

Working electrodes (12 μm nanocrystalline TiO_2 electrodes with a 4 μm scattering layer) were prepared and modified following the reported procedure.[4] The dye-loaded electrodes were prepared by dipping TiO_2 electrodes (0.3 cm × 0.4 cm) into a 0.3 mM dye solution ($CHCl_3$/EtOH = 1/1) for 12 h. To prepare the counter electrode, the Pt catalyst was deposited on the cleaned FTO glass by spin coating with a drop of H_2PtCl_6 solution (0.02 M in 2-propanol solution) with the heat treatment at 500 °C for 30 min. A hole (0.8 mm diameter) was predrilled on the counter electrode with a drill press. The two electrodes were sandwiched using a 25 μm thick hot-melt gasket. In this work, 0.5 M 1-butyl-3-methylimidiazolium iodide (BMII), 0.1 M 1,2-dimethyl-3-propylimidazolium iodide (DMPII), 0.1 M LiI, 0.05 M I_2, 0.1 M guanidinium thiocyanate (GuSCN) and 0.5 M *tert*-butylpyridine (TBP) in 85/15 mixture of acetonitrile and valeronitrile were used as the redox electrolyte.

4. Photovoltaic characterization

Photocurrent density−voltage (I−V) curves were obtained by illuminating the cell through the FTO substrate from the photoanode side under standard AM 1.5 conditions with a model 2400 source meter (Keithley Instruments, Inc. USA). The power of the simulated light was calibrated to 100 mW cm^{-2} using a Newport Oriel PV reference cell system (model 91150 V). The photocurrent action spectra were measured with Newport-74125 system (Newport Instruments). The intensity of the monochromic light was calibrated by a reference silicon cell (Newport-71640), and the aperture area of the employed metal mask is 0.160 cm^{-2}. The surface morphology of TiO_2 film was investigated by a Hitachi S-4800 Scanning Electron Microscope (SEM).

5. Electrochemical impedance spectroscopy (EIS)

Electrochemical impedance spectroscopy (EIS) for DSSCs was performed using a two-electrode system under dark with electrochemical workstation (Zahner IM6e). The spectra were scanned in a frequency range of 0.1 Hz - 100 kHz at room temperature under a series of applied bias potential with a magnitude of the alternative signal of 10 mV and characterized using Z-View software (Solartron Analytical).

References

[S1] W. Li, B. Liu, Y. Wu, S. Zhu, Q. Zhang, W. Zhu, *Dyes Pigments* **2013**, *99*, 176-184.

[S2] W. Zhu, Y. Wu, S. Wang, W. Li, X. Li, J. Chen, Z. Wang, H. Tian, *Adv. Funct. Mater.* **2011**, *21*, 756-763

[S3] Z. Wang, M. Liang, L. Wang, Y. Hao, C. Wang, Z. Sun, S. Xue, *Chem. Commun.* **2013**, *49*, 5748-5750.

[S4] S. Ito, T. N. Murakami, P. Comte, P. Liska, C. Grätzel, M. K. Nazeeruddin, M. Grätzel, *Thin Solid Films* **2008**, *516*, 4613-4619.

6. SEM spectra of TiO_2 film

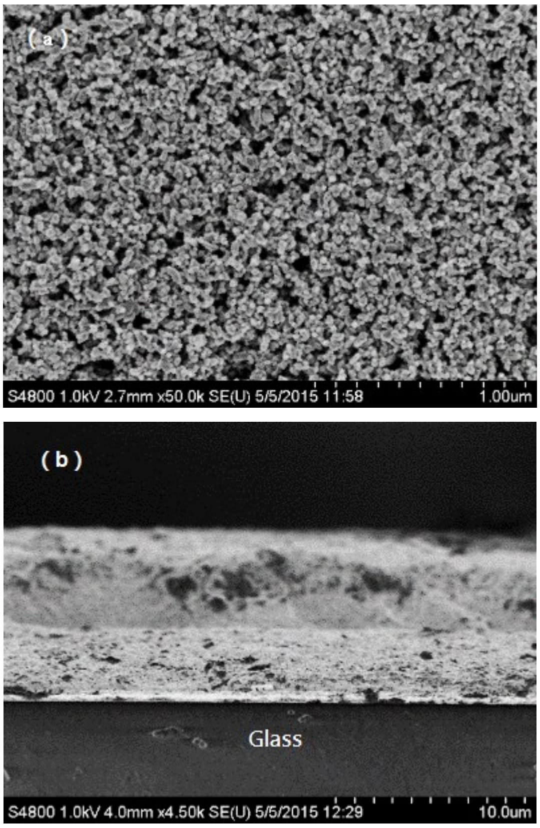

Figure S1. SEM of the plane-view (a) and cross-section (b) morphology image of the TiO_2 film. Note: The plane-view (a) clearly shows the TiO_2 film uniformly formed on the surface of FTO glass with a porous structure. This structure, not only provides enough adsorption sites for dyes, but also ensures the free diffusion of electrolyte and the regeneration of oxidation state dyes. The cross-section (b) presents the hamburger structure of photoanode (glass + FTO + transparent layer TiO_2 film + scattering layer TiO_2 film). At same time, it indicates the thickness of the transparent layer and scattering layer are around 4 μm, respectively.

7. IPCE spectra of WS-52, WS-53, WS-54 and WS-55, and their corresponding integrated current curves

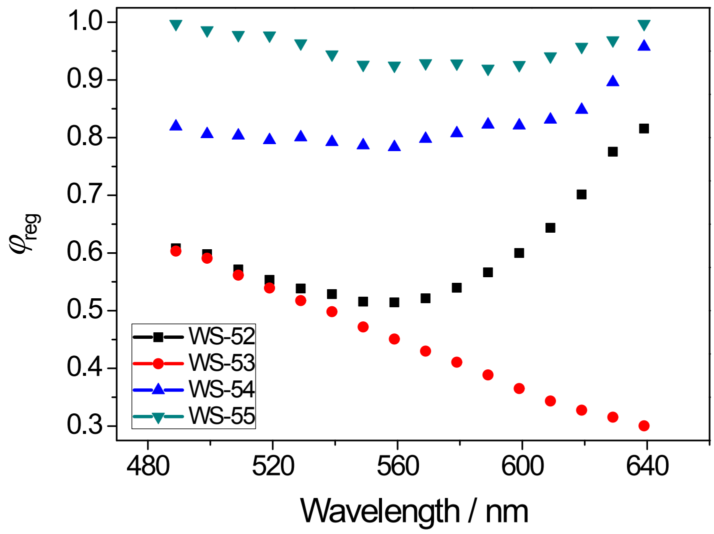

Figure S2. Calculated the dye regeneration efficiency (φ_{reg}) curves *vs.* wavelength based on the formula (IPCE = LHE × φ_{inj} × φ_{reg} × η_{coll}), where LHE is the light-harvesting efficiency related to the incident light absorbed by dye molecules, φ_{inj} the electron injection efficiency from the excited dye molecules into TiO_2 conduction band, φ_{reg} the dye regeneration efficiency, and η_{coll} the collection efficiency of injected electrons to the FTO substrate.

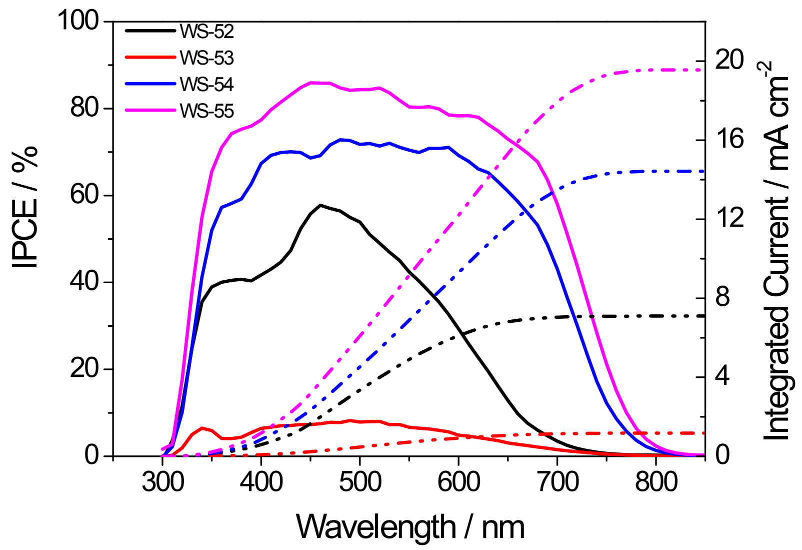

Figure S3. (Above) IPCE spectra of **WS-52**, **WS-53, WS-54** and **WS-55** (solid line), and their corresponding integrated current curves (dash dot line). Note: the integrated current for different dyes **WS-52**, **WS-53**, **WS-54** and **WS-54** are 7.10, 1.17, 14.43 and 19.56 mA cm^{-2}, respectively, which are in good agreement with the measured photocurrent J_{SC} (7.88, 1.22, 15.84 and 19.66 mA cm^{-2}).

8. The device stability character of dye WS-55

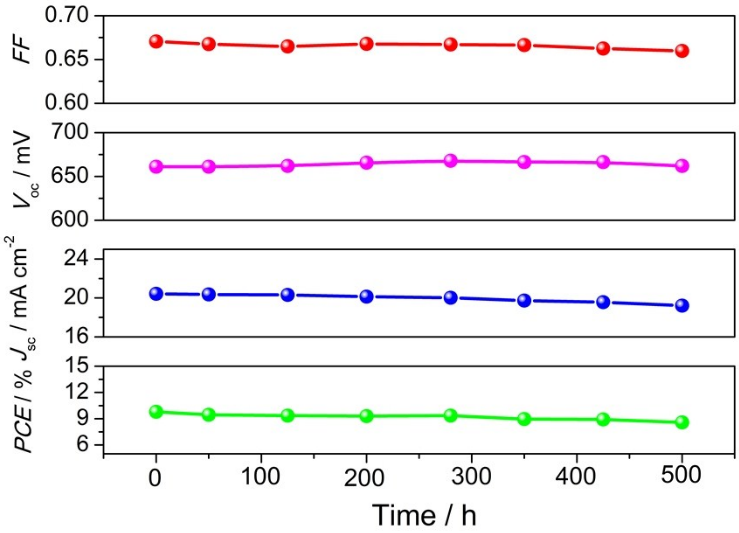

Figure S4. Variations of the photovoltaic parameters (*FF*, V_{oc}, J_{sc} and *PCE*) with aging time for the DSSC device based on **WS-55** under visible-light soaking.

9. The electrochemical impedance spectroscopy

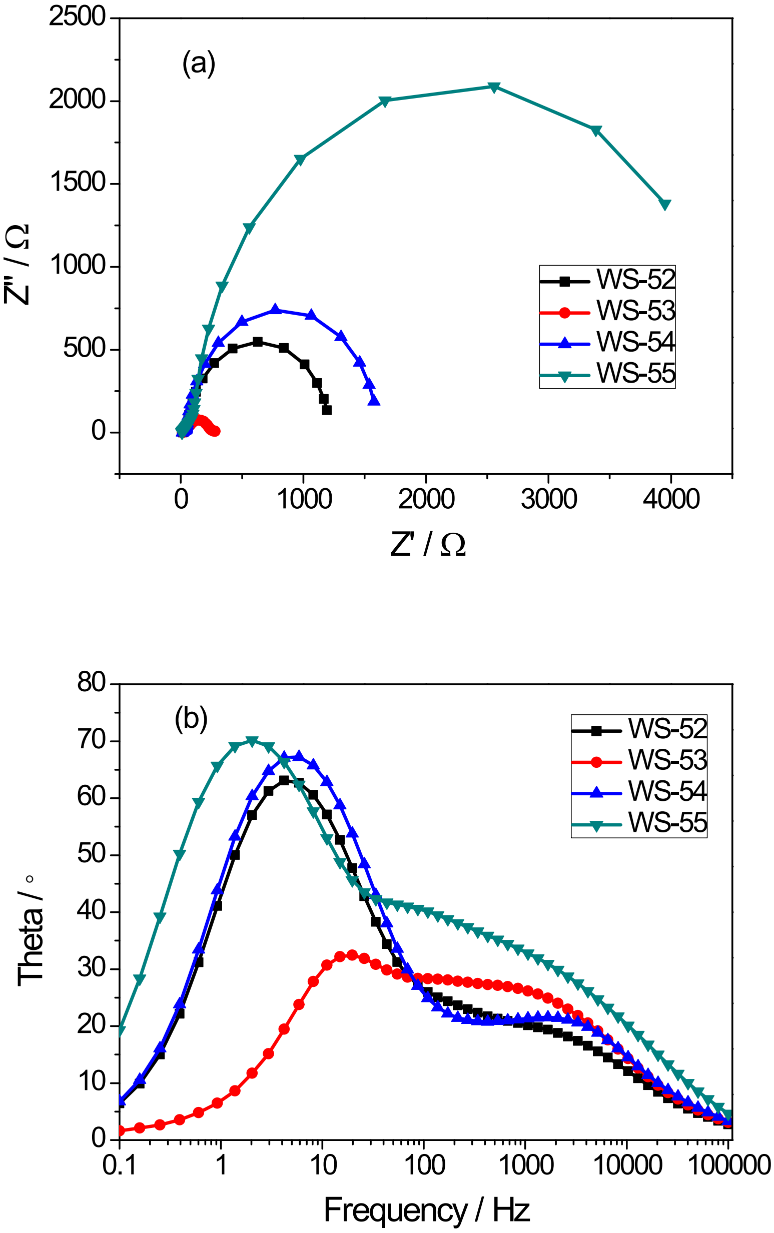

Figure S5. Impedance spectra of DSSCs based on **WS-52**, **WS-53**, **WS-54** and **WS-55** measured at 0.55 V bias in the dark. (a) Nyquist plots; (b) Bode phase plots.

10. Photovoltaic data of different DSSC samples based on WS-55

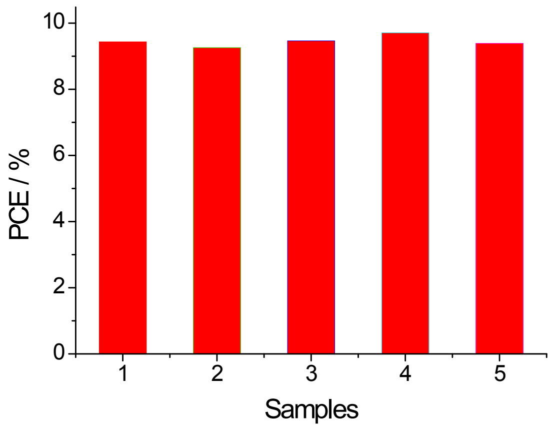

Samples	J_{sc}(mA cm^{-2})	V_{oc} (mV)	*FF*	*PCE* (%)
1	19.66	678	0.71	9.44
2	18.73	672	0.75	9.39
3	19.83	666	0.72	9.51
4	19.67	679	0.73	9.75
5	19.88	669	0.69	9.23

Figure S6. Photovoltaic data of different DSSC samples based on **WS-55**.

11. Photoelectric parameters (J_{SC}, V_{OC} and PCE) under different light intensity based on Dye WS-55

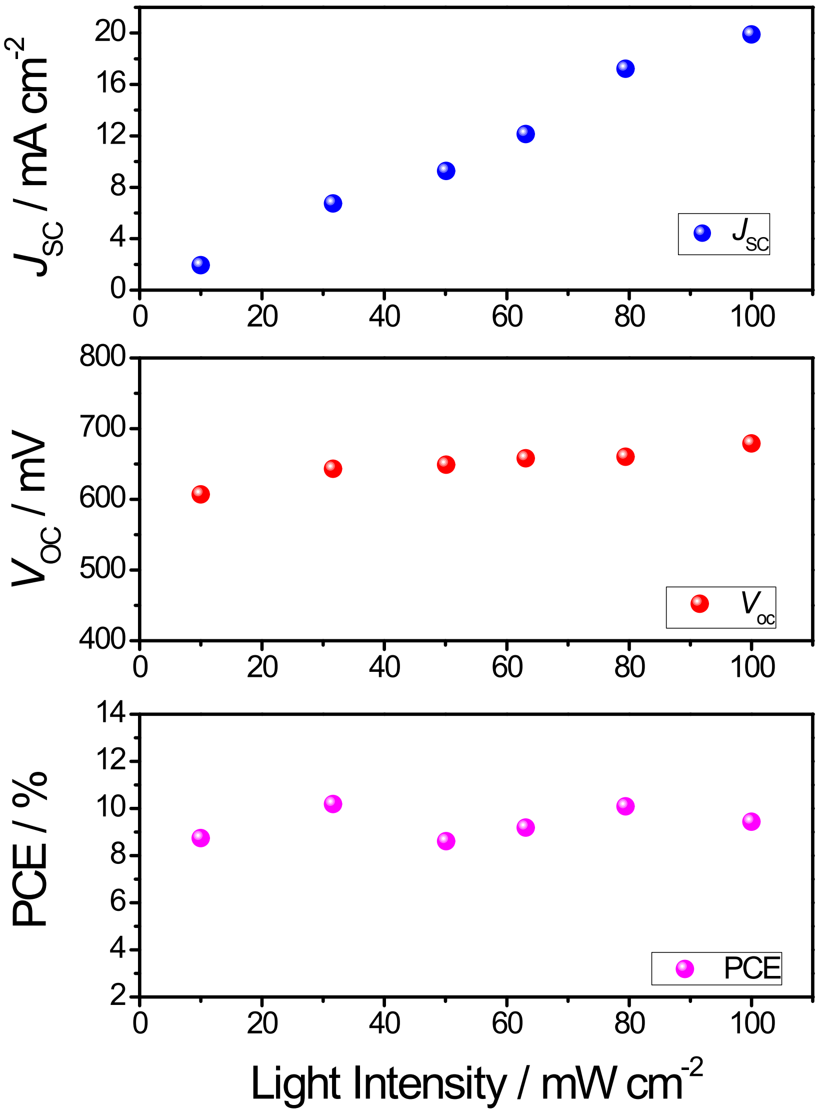

Figure S7. Photoelectric parameters (J_{SC}, V_{OC} and PCE) under different light intensity based on Dye **WS-55**.

12. ^{1}H NMR, ^{13}C NMR and HRMS of intermediates and targeted dyes

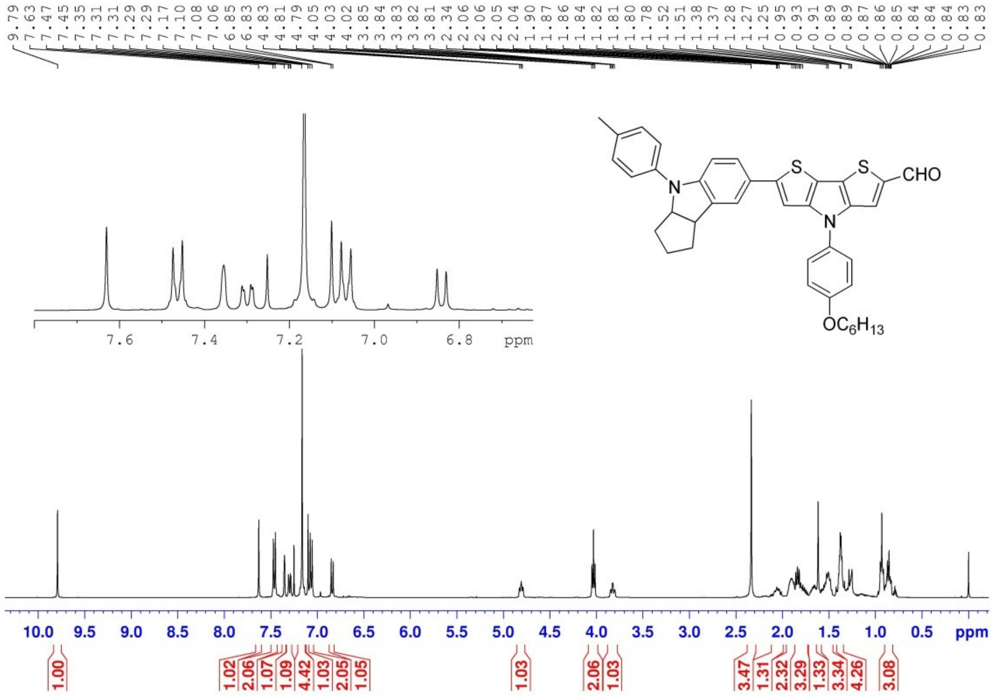

Figure S8. ^{1}H NMR of compound **4** in $CDCl_3$

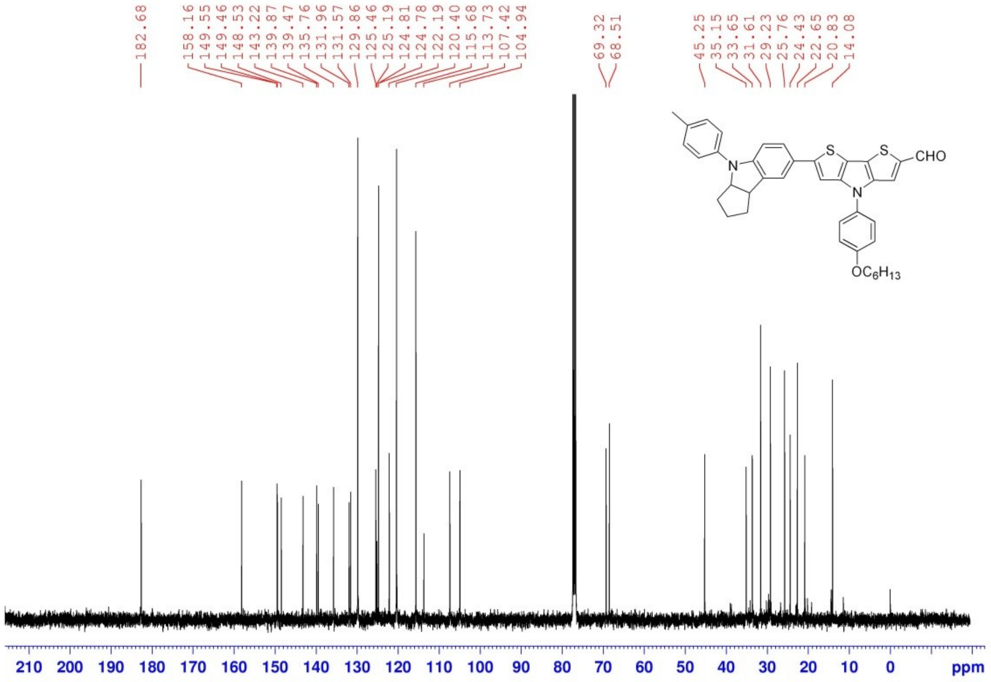

Figure S9. ^{13}C NMR of compound **4** in $CDCl_3$

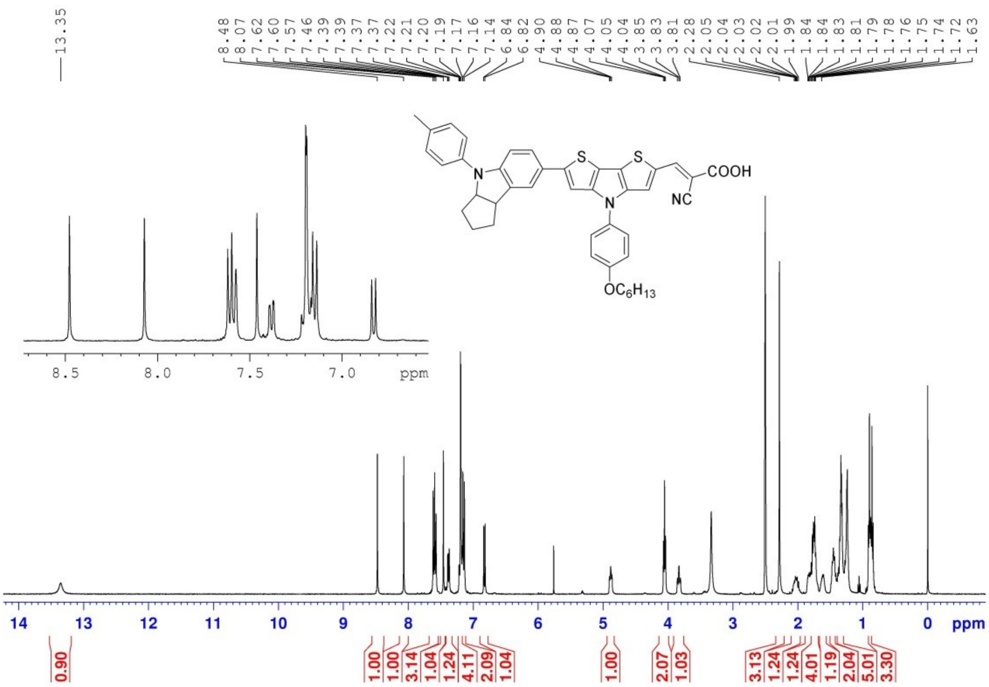

Figure S10. ^{1}H NMR of **WS-52** in DMSO

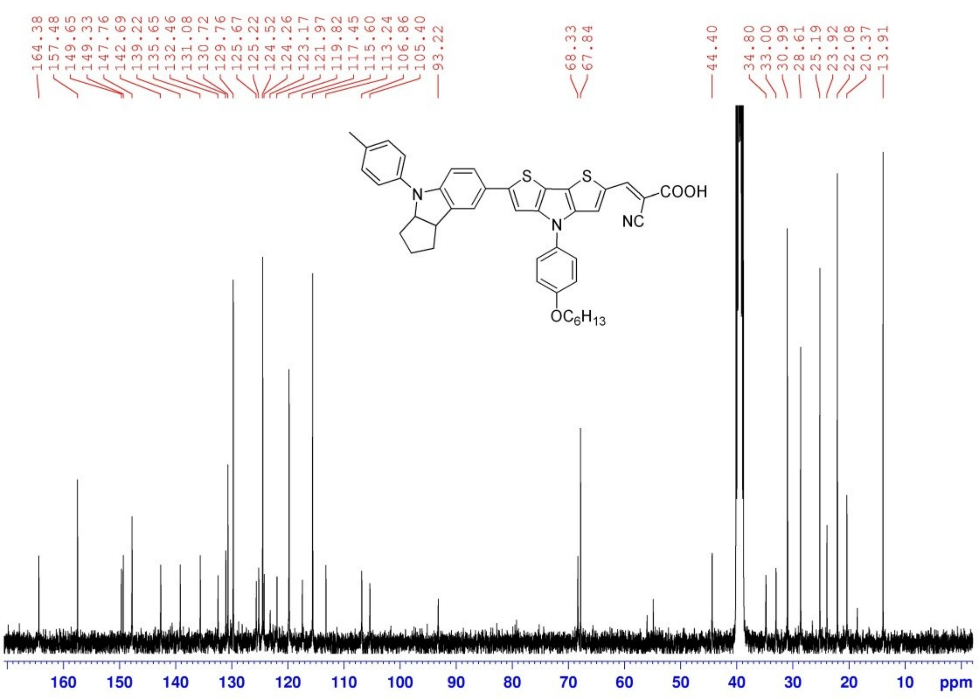

Figure S11. ^{13}C NMR of **WS-52** in DMSO

Elemental Composition Report **Page 1**

Single Mass Analysis
Tolerance = 30.0 mDa / DBE: min = -1.5, max = 100.0
Element prediction: Off
Number of isotope peaks used for i-FIT = 2

Monoisotopic Mass, Odd and Even Electron Ions
36 formula(e) evaluated with 1 results within limits (up to 1 best isotopic matches for each mass)
Elements Used:
C: 0-42 H: 0-50 N: 0-3 O: 0-3 S: 0-2

WH-ZHU ECUST institute of Fine Chem 12-Jan-2015 20:06:53

ZW-ZH-62 114 (0.791) Cm (110:116) 1: TOF MS ES+ 1.48e+004

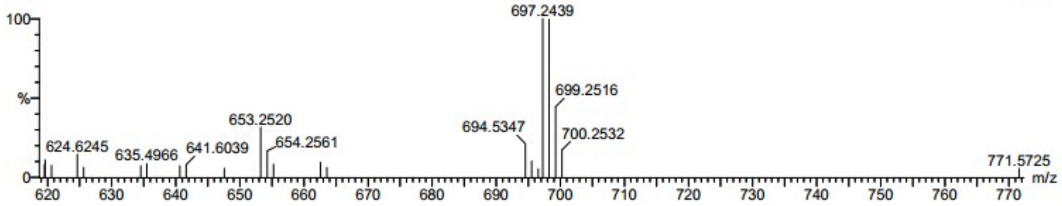

Minimum:				-1.5			
Maximum:		30.0	50.0	100.0			
Mass	Calc. Mass	mDa	PPM	DBE	i-FIT	i-FIT (Norm)	Formula
697.2439	697.2433	0.6	0.9	25.0	23.7	0.0	C42 H39 N3 O3 S2

Figure S12. HRMS of **WS-52**

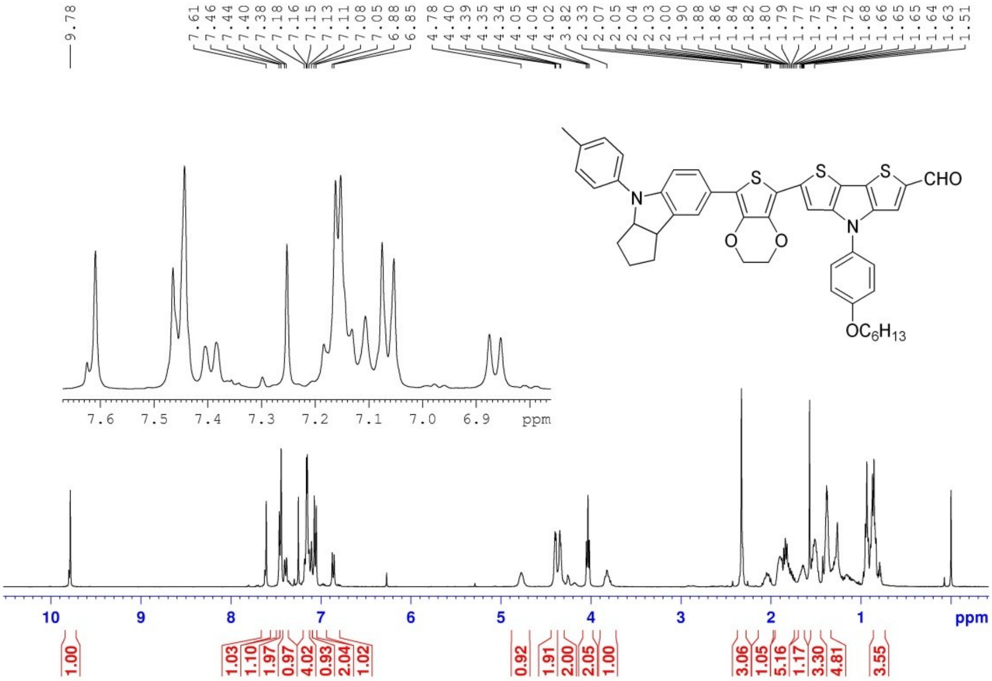

Figure S13. ^{1}H NMR of compound **5** in $CDCl_3$

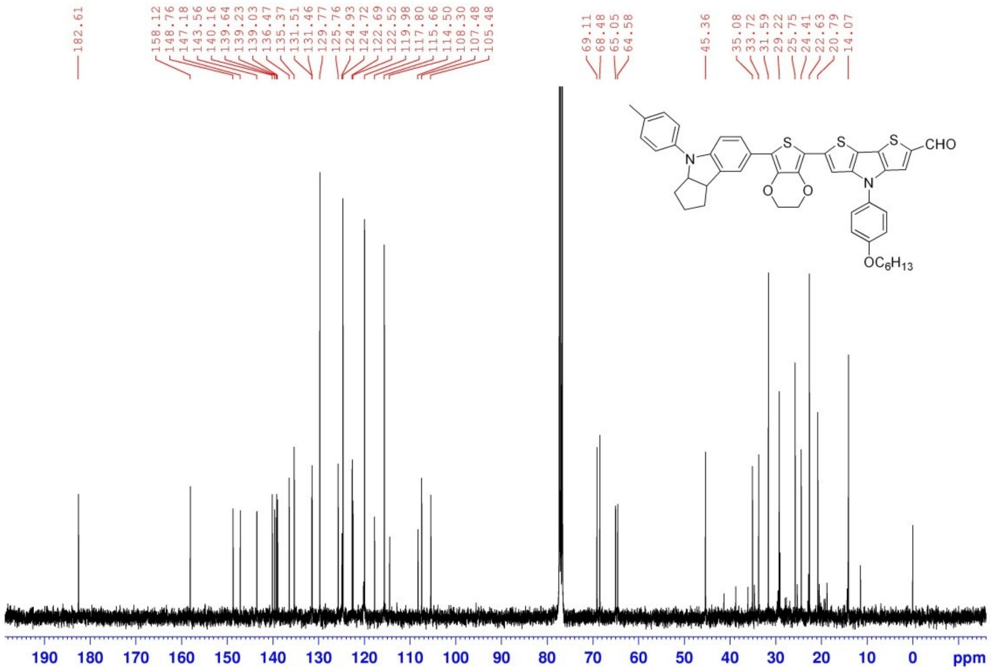

Figure S14. ^{13}C NMR of compound **5** in $CDCl_3$

Elemental Composition Report **Page 1**

Single Mass Analysis
Tolerance = 30.0 mDa / DBE: min = -1.5, max = 100.0
Element prediction: Off
Number of isotope peaks used for i-FIT = 2

Monoisotopic Mass, Odd and Even Electron Ions
47 formula(e) evaluated with 1 results within limits (up to 1 closest results for each mass)
Elements Used:
C: 0-45 H: 0-50 N: 0-2 O: 0-4 S: 0-3

WH-ZHU ECUST institute of Fine Chem 31-Oct-2013 18:58:08

ZW-ZHB-076 108 (0.761) Cm (91:108) 1: TOF MS ES+ 5.21e+003

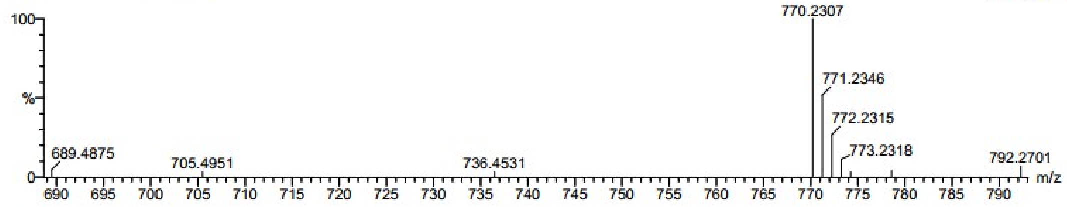

Minimum:				-1.5			
Maximum:		30.0	50.0	100.0			
Mass	Calc. Mass	mDa	PPM	DBE	i-FIT	i-FIT (Norm)	Formula
770.2307	770.2307	0.0	0.0	26.0	9.1	0.0	C45 H42 N2 O4 S3

Figure S15. HRMS of compound **5**

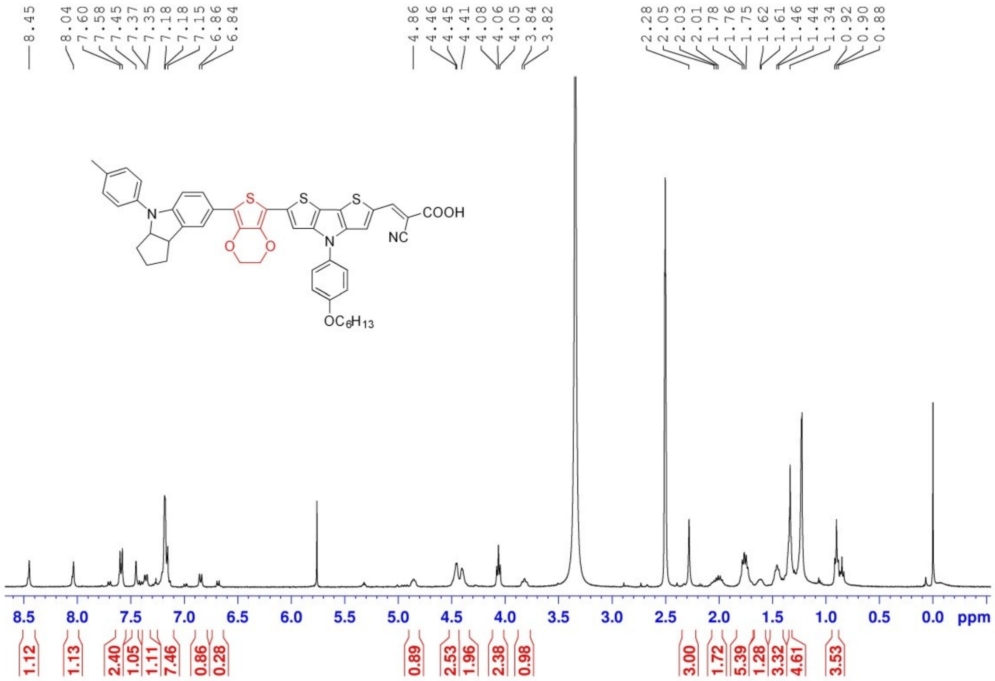

Figure S16. ^{1}H NMR of **WS-53** in DMSO

Elemental Composition Report **Page 1**

Single Mass Analysis
Tolerance = 30.0 mDa / DBE: min = -1.5, max = 100.0
Element prediction: Off
Number of isotope peaks used for i-FIT = 2

Monoisotopic Mass, Odd and Even Electron Ions
69 formula(e) evaluated with 3 results within limits (up to 1 closest results for each mass)
Elements Used:
C: 0-50 H: 0-60 N: 0-3 O: 0-5 S: 0-3

WH-ZHU ECUST institute of Fine Chem 02-Nov-2013 13:53:21

ZWH-ZHB-077 20 (0.691) Cm (18:24) 1: TOF MS ES+ 6.46e+003

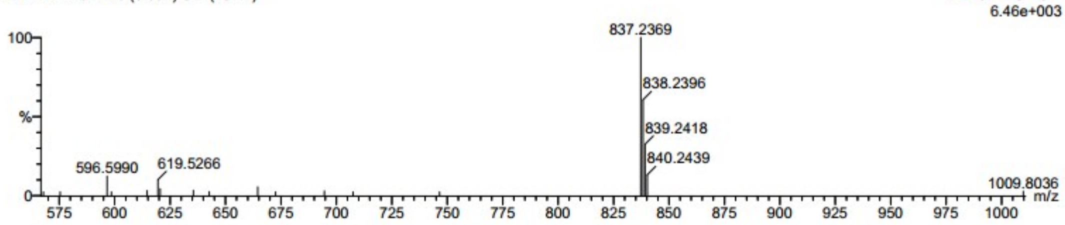

Minimum:				-1.5			
Maximum:		30.0	50.0	100.0			
Mass	Calc. Mass	mDa	PPM	DBE	i-FIT	i-FIT (Norm)	Formula
837.2369	837.2365	0.4	0.5	29.0	9.5	0.0	C48 H43 N3 O5 S3

Figure S17. HRMS of compound **WS-53**

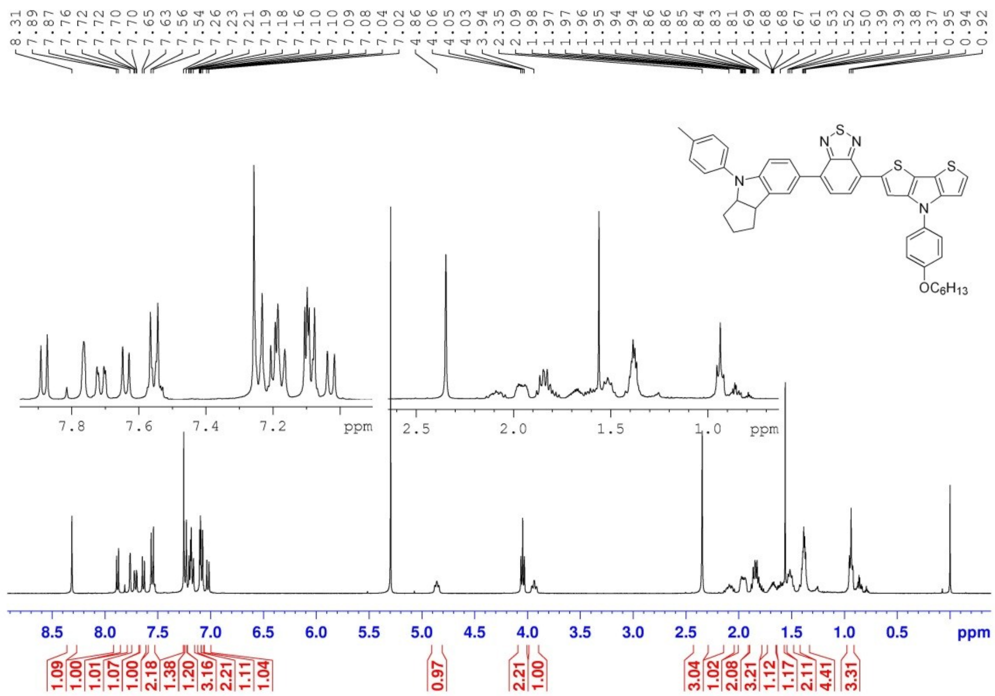

Figure S18. ^{1}H NMR of compound **8** in $CDCl_3$

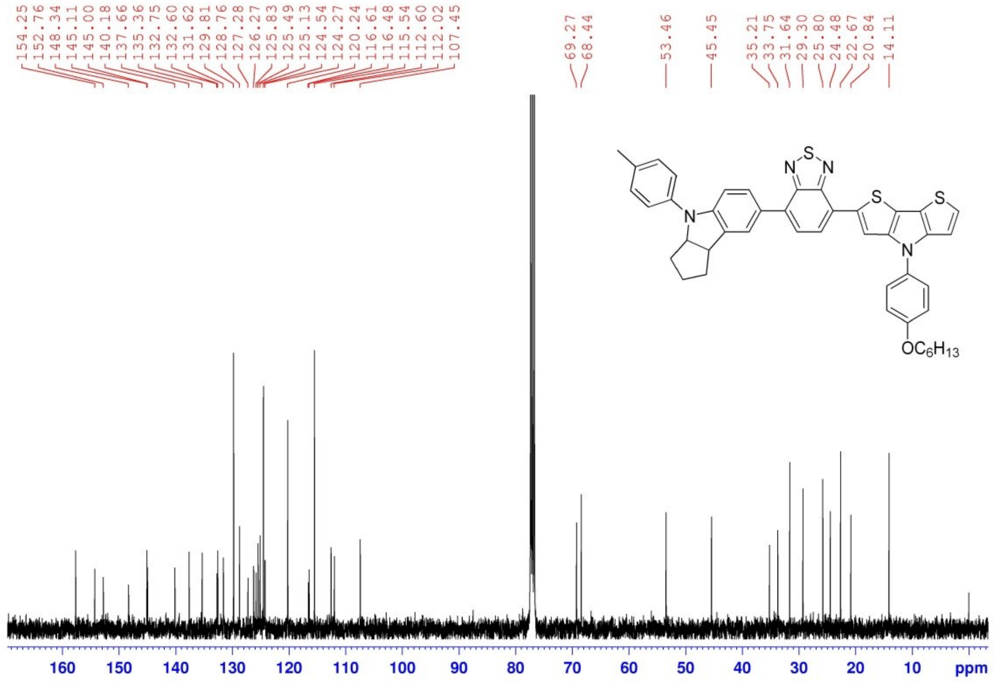

Figure S19. ^{13}C NMR of compound **8** in $CDCl_3$

Elemental Composition Report Page 1

Single Mass Analysis
Tolerance = 30.0 mDa / DBE: min = -1.5, max = 100.0
Element prediction: Off
Number of isotope peaks used for i-FIT = 2

Monoisotopic Mass, Even Electron Ions
59 formula(e) evaluated with 6 results within limits (up to 1 closest results for each mass)
Elements Used:
C: 0-50 H: 0-50 N: 0-4 O: 0-1 S: 0-3

WH-ZHU ECUST institute of Fine Chem 05-Jan-2014 13:09:43

ZW-ZHB-1 56 (0.441) Cm (55:63) 1: TOF MS ES+ 5.21e+003

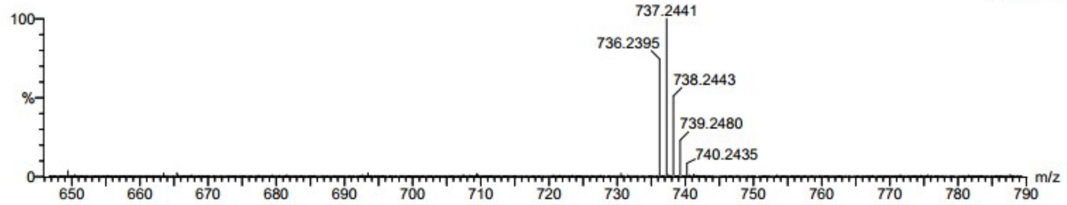

Minimum:				-1.5			
Maximum:		30.0	50.0	100.0			
Mass	Calc. Mass	mDa	PPM	DBE	i-FIT	i-FIT (Norm)	Formula
737.2441	737.2443	-0.2	-0.3	26.5	77.7	0.0	C44 H41 N4 O S3

Figure S20. HRMS of compound **8**

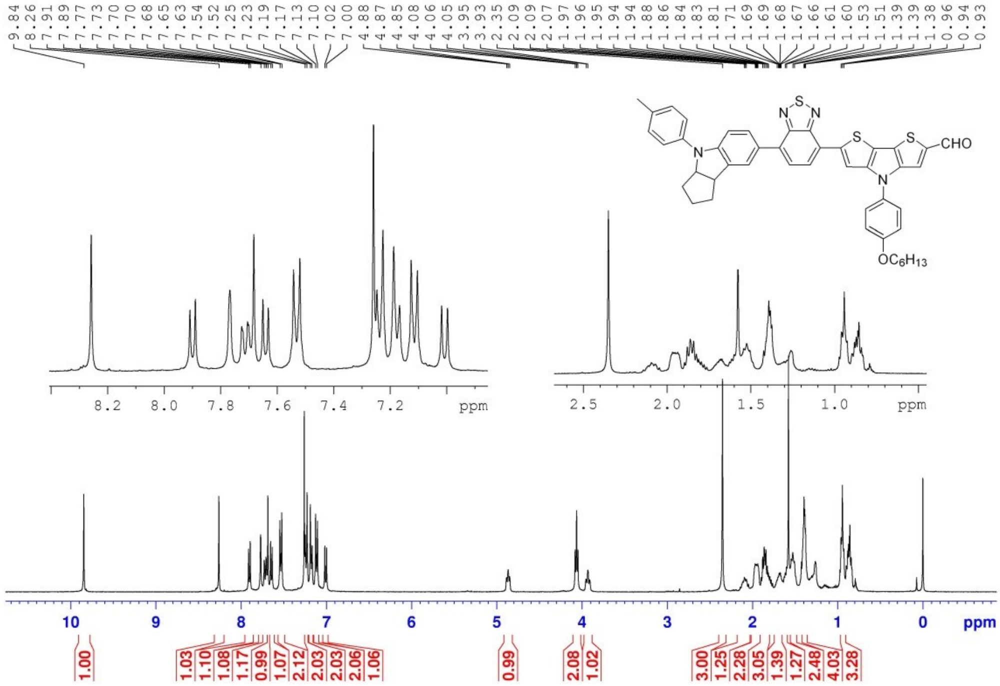

Figure S21. ^{1}H NMR of compound **9** in $CDCl_3$

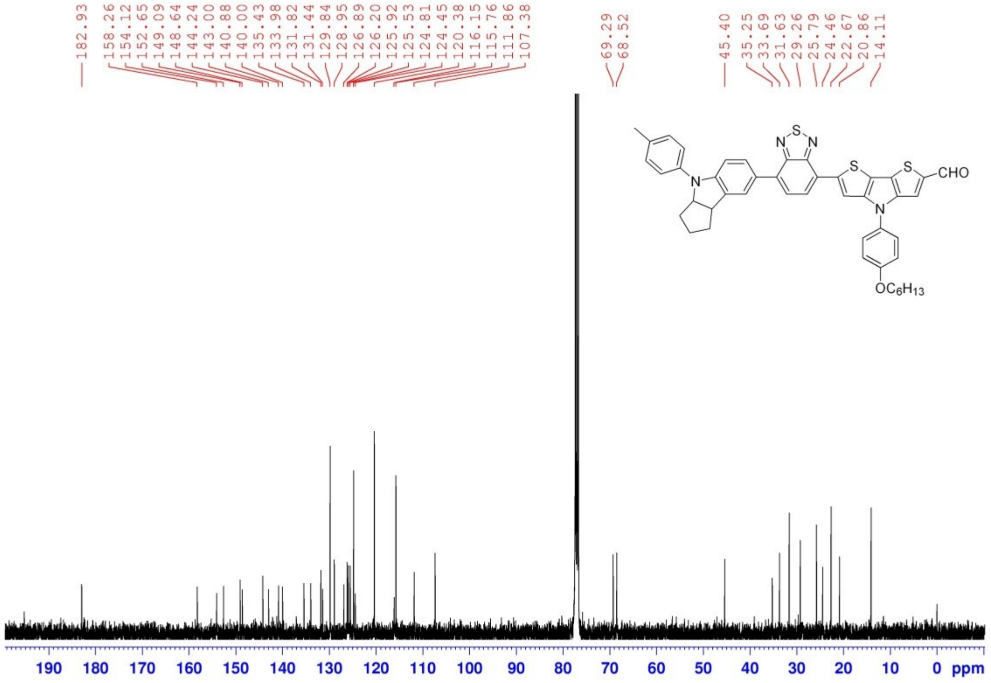

Figure S22. ^{13}C NMR of compound **9** in $CDCl_3$

Elemental Composition Report **Page 1**

Single Mass Analysis
Tolerance = 30.0 mDa / DBE: min = -1.5, max = 100.0
Element prediction: Off
Number of isotope peaks used for i-FIT = 2

Monoisotopic Mass, Even Electron Ions
96 formula(e) evaluated with 9 results within limits (up to 1 closest results for each mass)
Elements Used:
C: 0-50 H: 0-50 N: 0-4 O: 0-3 S: 0-3
WH-ZHU ECUST institute of Fine Chem 11-Jan-2014 12:20:23
ZWH-ZHB-1011 6 (0.275) Cm (6) 1: TOF MS ES+ 1.16e+002

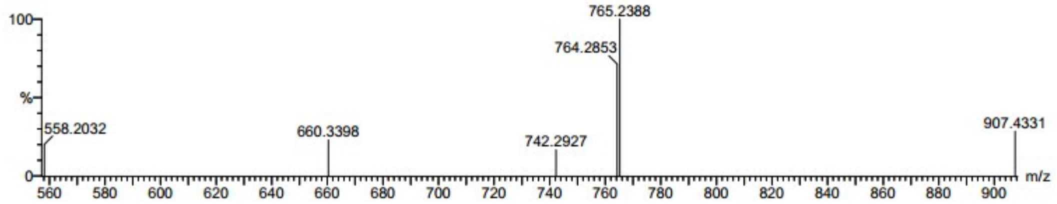

Minimum:				-1.5			
Maximum:		30.0	50.0	100.0			
Mass	Calc. Mass	mDa	PPM	DBE	i-FIT	i-FIT (Norm)	Formula
765.2388	765.2392	-0.4	-0.5	27.5	10.6	0.0	C45 H41 N4 O2 S3

Figure S23. HRMS of compound **9**

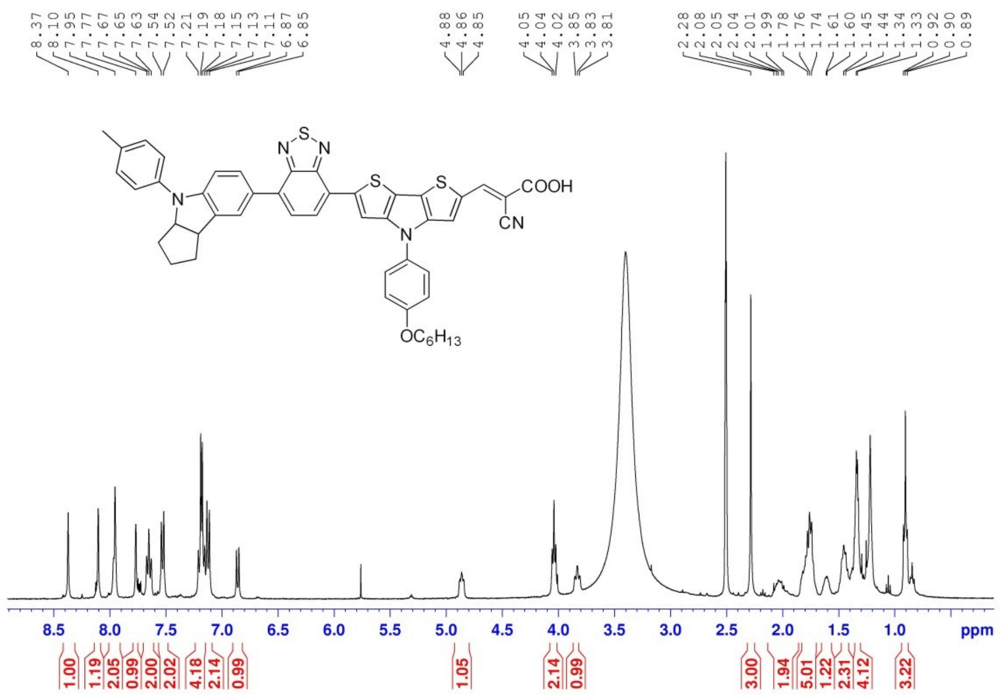

Figure S24. ^{1}H NMR of **WS-54** in DMSO

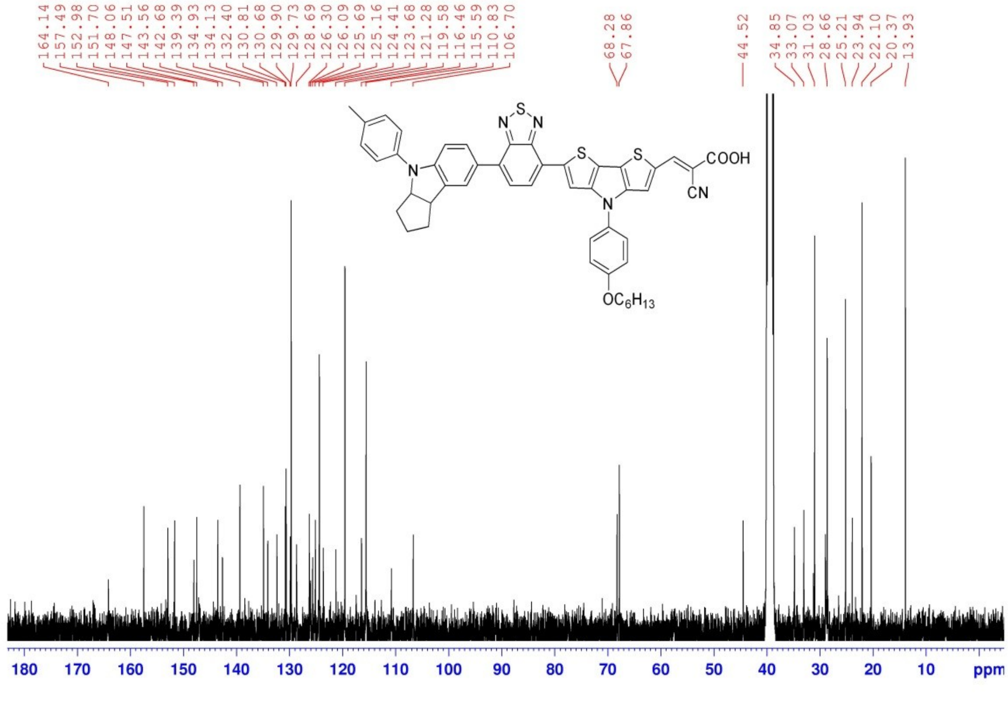

Figure S25. ^{13}C NMR of **WS-54** in DMSO

Elemental Composition Report **Page 1**

Single Mass Analysis
Tolerance = 30.0 mDa / DBE: min = -1.5, max = 100.0
Element prediction: Off
Number of isotope peaks used for i-FIT = 2

Monoisotopic Mass, Even Electron Ions
76 formula(e) evaluated with 1 results within limits (up to 1 best isotopic matches for each mass)
Elements Used:
C: 0-48 H: 0-50 N: 0-5 O: 0-3 S: 0-3

WH-ZHU ECUST institute of Fine Chem 12-Jan-2015 20:01:46

ZW-ZH-64 273 (1.770) Cm (267:274) 1: TOF MS ES+ 5.97e+003

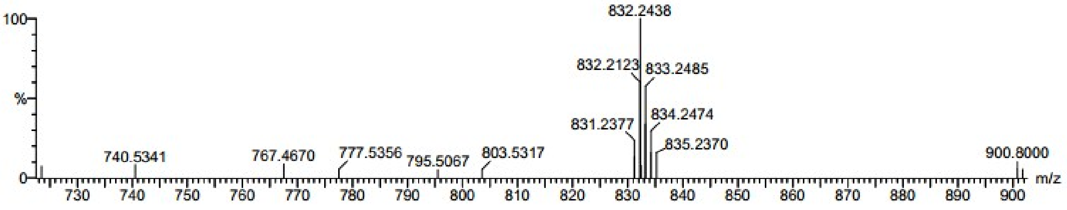

Minimum:				-1.5			
Maximum:		30.0	50.0	100.0			
Mass	Calc. Mass	mDa	PPM	DBE	i-FIT	i-FIT (Norm)	Formula
832.2438	832.2450	-1.2	-1.4	30.5	36.5	0.0	C48 H42 N5 O3 S3

Figure S26. HRMS of **WS-54**

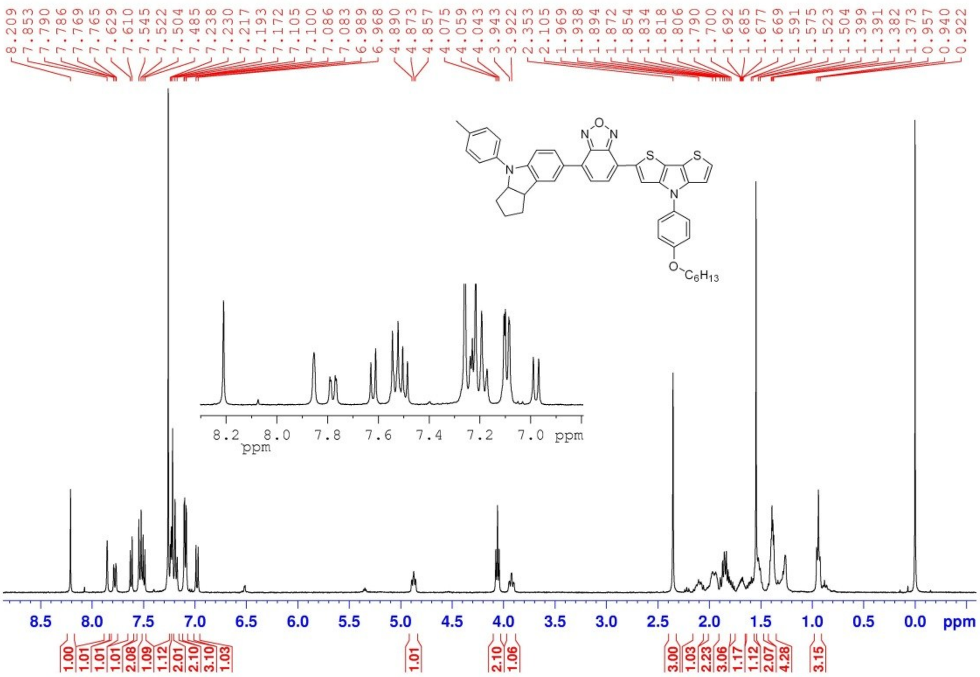

Figure S27. ^{1}H NMR of compound **11** in $CDCl_3$

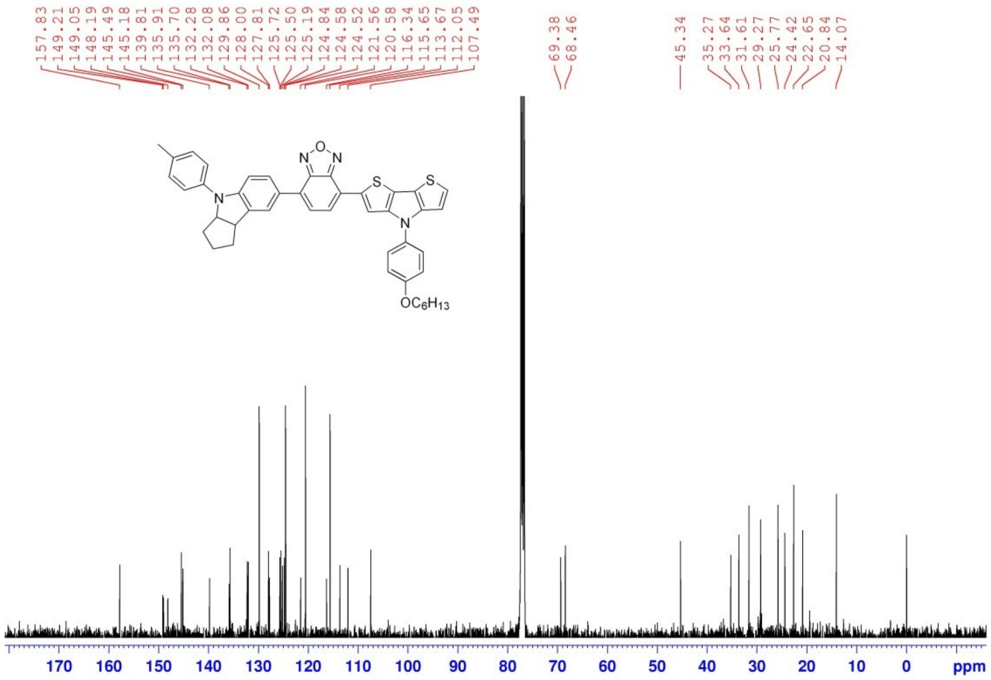

Figure S28. ^{13}C NMR of compound **11** in $CDCl_3$

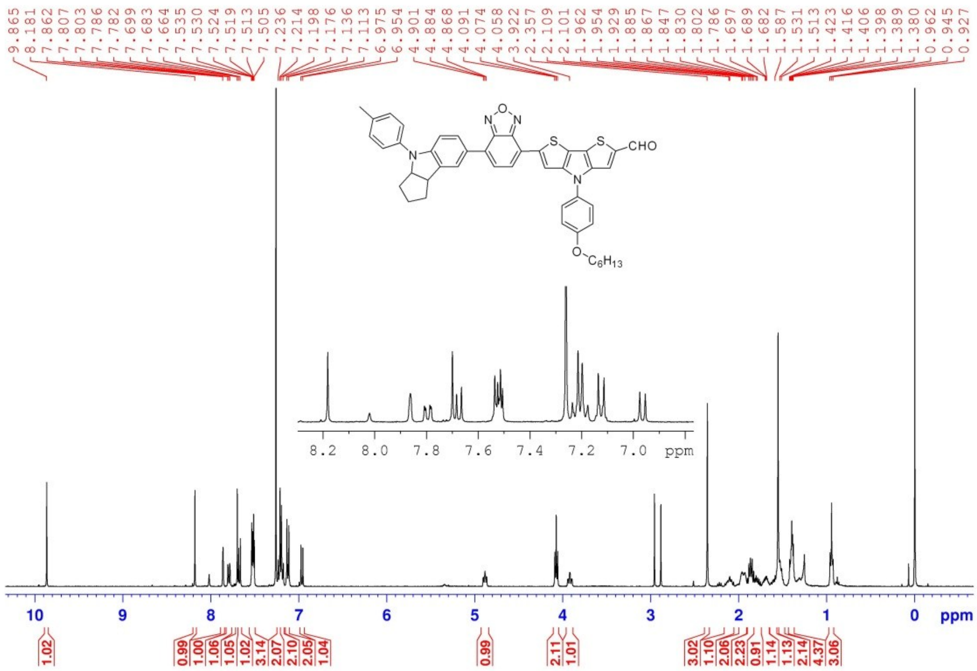

Figure S29. ^{1}H NMR of compound **12** in $CDCl_3$

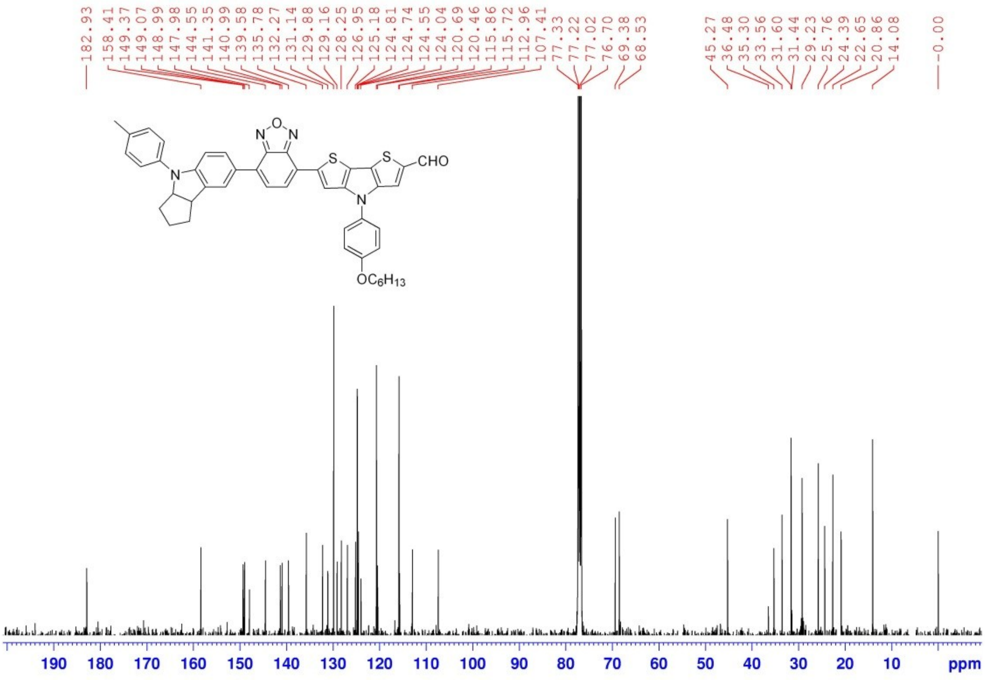

Figure S30. ^{13}C NMR of compound **12** in $CDCl_3$

Elemental Composition Report **Page 1**

Single Mass Analysis

Tolerance = 50.0 PPM / DBE: min = -1.5, max = 100.0

Element prediction: Off

Number of isotope peaks used for i-FIT = 2

Monoisotopic Mass, Even Electron Ions

58 formula(e) evaluated with 10 results within limits (up to 1 best isotopic matches for each mass)

Elements Used:

C: 0-50 H: 0-50 N: 0-4 O: 0-3 S: 0-2

WH-ZHU ECUST institute of Fine Chem 26-Mar-2015 20:48:33

ZWH-ZWW-1 90 (0.645) Cm (89:91) 1: TOF MS ES+ 2.36e+003

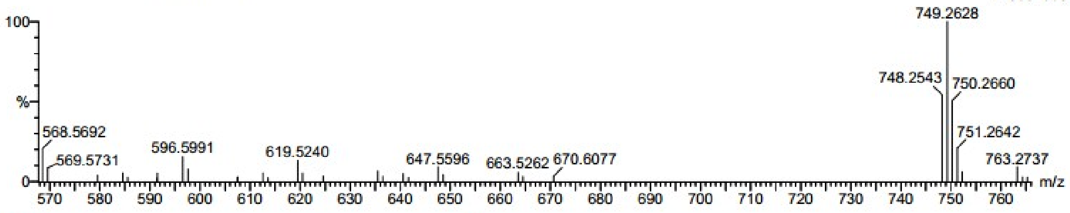

Minimum:				-1.5			
Maximum:		300.0	50.0	100.0			
Mass	Calc. Mass	mDa	PPM	DBE	i-FIT	i-FIT (Norm)	Formula
749.2628	749.2620	0.8	1.1	27.5	8.6	0.0	C45 H41 N4 O3 S2

Figure S31. HRMS of compound **12**

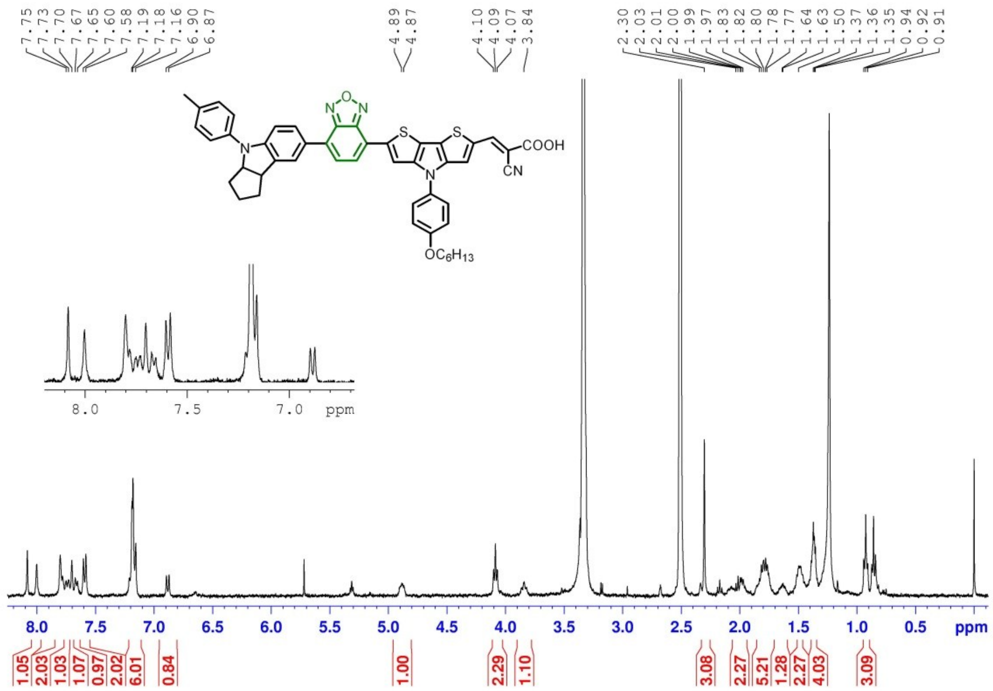

Figure S32. ^1H NMR of **WS-55** in DMSO and $CDCl_3$

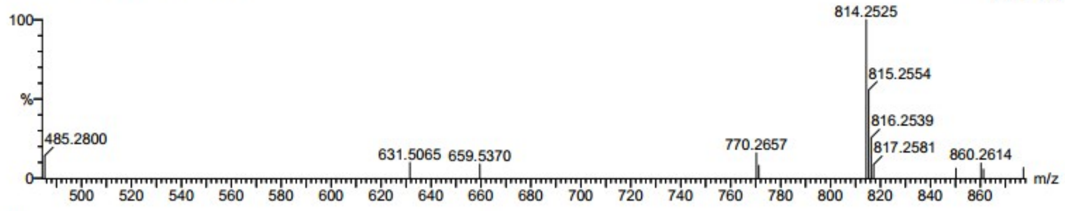

Elemental Composition Report Page 1

Single Mass Analysis
Tolerance = 50.0 PPM / DBE: min = -1.5, max = 100.0
Element prediction: Off
Number of isotope peaks used for i-FIT = 2

Monoisotopic Mass, Even Electron Ions
51 formula(e) evaluated with 1 results within limits (up to 1 best isotopic matches for each mass)
Elements Used:
C: 0-50 H: 0-42 N: 0-5 O: 0-4 S: 0-2
WH-ZHU ECUST institute of Fine Chem 26-Mar-2015 20:54:09
ZWH-ZWW-2 120 (2.710) Cm (118:121) 1: TOF MS ES- 8.16e+003

Minimum:				-1.5			
Maximum:		300.0	50.0	100.0			
Mass	Calc. Mass	mDa	PPM	DBE	i-FIT	i-FIT (Norm)	Formula
814.2525	814.2522	0.3	0.4	31.5	9.6	0.0	C48 H40 N5 O4 S2

Figure S33. HRMS of compound **WS-55**
